# Analytical Methodology for a Metabolome Atlas of Goat’s Plasma, Milk and Feces Using ^1^H-NMR and UHPLC-HRMS

**DOI:** 10.3390/metabo11100681

**Published:** 2021-10-04

**Authors:** Cécile Martias, Julie Gatien, Léa Roch, Nadine Baroukh, Sylvie Mavel, Antoine Lefèvre, Frédéric Montigny, Laurent Schibler, Patrick Emond, Lydie Nadal-Desbarats

**Affiliations:** 1 UMR 1253 iBrain, University of Tours, Inserm, 37044 Tours, France; cecile.martias@univ-tours.fr (C.M.); nadine.baroukh@univ-tours.fr (N.B.); sylvie.mavel@univ-tours.fr (S.M.); antoine.lefevre@univ-tours.fr (A.L.); frederic.montigny@univ-tours.fr (F.M.); patrick.emond@univ-tours.fr (P.E.); 2 Allice, Phenotyping Station, 37380 Nouzilly, France; julie.gatien@allice.fr (J.G.); lroch8@gmail.com (L.R.); laurent.schibler@allice.fr (L.S.); 3 CHRU Tours, Medical Biology Center, 37000 Tours, France

**Keywords:** metabolic fingerprinting, ^1^H-NMR, LC-MS, plasma, milk, feces, goat

## Abstract

Metabolomics has been increasingly used in animal and food sciences. Animal health is one of the most important factor that can also alter animal integrity and welfare. Some studies have already investigated the link between health and metabolic profile of dairy animals. These studies in metabolomics often consider a single type of sample using a single analytical platform (nuclear magnetic resonance or mass spectrometry). Only few studies with multi-platform approaches are also used with a single or a multi type of sample, but they mainly consider dairy cows’ metabolome although dairy goats present similar diseases, that it could be interesting to detect early to preserve animal health and milk production. This study aims to create a metabolic atlas of goat plasma, milk and feces, based on healthy animals. Our study describes a standard operating procedure for three goat matrices: blood plasma, milk, and feces using multiple platforms (NMR (^1^H), UHPLC (RP)-MS and UHPLC (HILIC)-MS) that follows a unique sample preparation procedure for each sample type to be analyzed on multi-platforms basis. Our method was evaluated for its robustness and allowed a better characterization of goat metabolic profile in healthy conditions.

## 1. Introduction

Over the last decades, animal health and welfare have generated a growing interest in livestock research [1]. The development of tools and identification of important new biomarkers have been a huge concern for the last twenty years to help breeders in the management of their herds. Animal health is a dominant factor influencing animal welfare, quality of products and farms’ income at the same time [2]. Nowadays, several parameters, such as rumination [3], activity, milk production and quality [4], are examined in routine by some farmers to monitor health of their animals, in addition to veterinarian diagnosis for proven diseases.

The study of dairy animals’ metabolome would allow better targeting biomarkers of diseases. Is nowadays well established that the metabolome represents the ultimate end-point of the biological ‘omics’ cascade. The metabolome is impacted by the complex interactions between the host and its microbiota and other factors such as diet, stress, gender or age. Metabolomics focuses on the wide variety of low molecular weight metabolites (<1500 Da) present in biological samples (cells, tissues or biological fluids) [5]. Metabolomics is now routinely used in biomedical and nutritional sciences [6]. Livestock metabolomics is an emerging area of metabolomics providing new biomarkers for early, rapid and non-invasive diagnosis in farm animals [7]. Applications of livestock metabolomics is conducted in different domains such as animal welfare assessment, disease diagnosis [8], biomarker discovery for diet efficiency [9,10], growth potential and milk production.

Diseases such as ketosis or mastitis affect dairy animals and milk production. Among dairy cows after calving, ketosis and subclinical ketosis are usually found related to negative energy balance [11]. Mastitis inflammation can either be clinical and is usually associated with clinical signs or subclinical with no clinical sign but with elevated somatic cell count affecting the milk quality. One of the most important and expensive disease in dairy goat production is mastitis [12]. Mastitis is an infection of the udder, caused by the most common pathogens coagulase-negative staphylococci (CNS) and *Staphylococcus aureus* (*S. aureus*). Mastitis can appear mainly during the first third of lactation. In small ruminants like goats, the prevalence of subclinical mastitis averages 5–30% [13]. Contrary to clinical mastitis which is easy to detect, animals suffering from subclinical mastitis are often difficult to identify due to the lack of reliable diagnostic methods. Even if some studies related links between somatic cells counts and subclinical mastitis [14,15], early and reliable diagnosis establishment at farm level is difficult due to the time between the different milk control analyzes, associated to the absence of others clear signs.

However looking at the literature, very few efforts focused on metabolomics characterization of healthy animals with the aim to identifying baseline metabolite coverage in different biofluids or tissues. These baseline survey values are necessary and very often needed before biomarker studies should be carried on. Nowadays most of the metabolomics studies on dairy animals are usually done on a single biological fluid (mostly blood or milk) and using only one analytical platform (either chromatography-mass spectroscopy (MS) or nuclear magnetic resonance (NMR)). These two platforms are the most widely used technologies. In the literature, milk is one of the most frequently used fluid in dairy animals, as it is easily accessible and produced in a huge amount. Therefore, the search for biomarkers is often carried out by the analysis of milk, for health applications and for milk composition and quality for example [16,17,18]. However, the information often remained limited to a unique sample type. Some studies showed correlation between milk and blood which help in the detection of others biomarkers [9]. In fact, blood is a matrix commonly used to assess markers of stress in animals. An example is the measurement of cortisol hormone a stress indicator. Cortisol metabolites in blood have been shown to be directly correlated to fecal cortisol metabolites in stressed animals [19]. In most cases, feces can be collected without invasive procedure and in large quantities. Most of the time when multiple fluids are used to describe biological systems, the information remained limited to a unique analytical platform or in contrary when investigating metabolome combining multi-analytical platforms, studies have focused on a single biofluid. However Xu et al. showed that combining data from milk and plasma using ^1^H-NMR and UHPLC-MS platforms permitted to understand link between energy balance and metabolic profiles [9].

Currently, there is no study associating metabolomics information from multiple matrices using multiple platforms for goat metabolome atlas in healthy condition to get the widest metabolite coverage to describe an individual. Thus the present work describes a unique standard operating procedure by biological matrices (milk, plasma and feces) to be analyzed by different platforms (UHPLC (RP and HILIC)-MS and NMR). The proposal of a metabolomics atlas for goat could open a new path for breeders in monitoring the welfare of their herd.

## 2. Results

In order to depict as closely as possible the metabolomics coverage of healthy goat, we looked for the best preparation protocol to describe an individual with the largest metabolic coverage meaning using three biological matrices (plasma, milk and feces) easy to harvest and different analytical platforms (^1^H-NMR, HILIC-MS and RPLC-MS). The aim of this study was to obtain a single preparation procedure able to be explored by ^1^H-NMR, (ESI+/ESI-)RP-LC-MS and (ESI+/ESI-)HILIC-LC-MS platforms. The metabolic information collected may have the best reproducibility in the extraction protocol, the largest metabolic coverage without any a priori in the selected metabolites.

### 2.1. Optimization of Extraction Protocol

#### 2.1.1. Plasma

**Figure 1:**
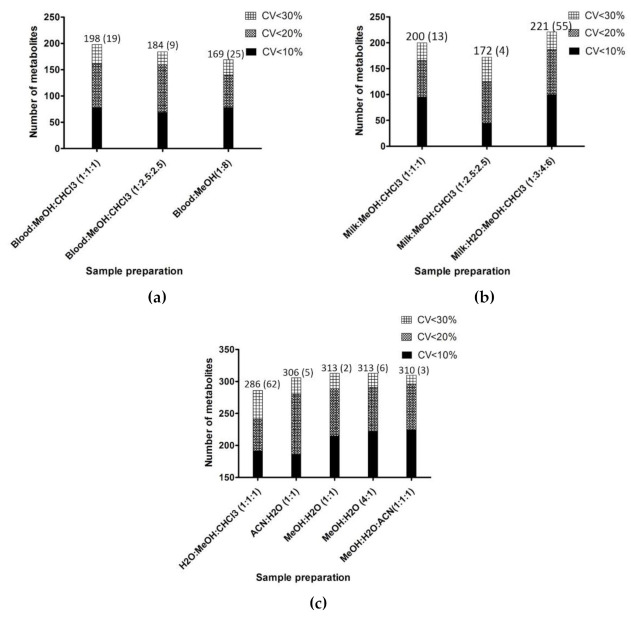
Histograms providing the total number of metabolites extracted (*n* = 10) for each extraction protocol and respectively their reproducibility evaluated by CV (Coefficient of Variation) in plasma (**a**), milk (**b**) and feces (**c**). In bracket, are mentioned the numbers of metabolites analyzed by only one extraction protocol.

A total of three preparation protocols were tested with plasma samples (see materials and methods Figure S1a), two biphasic extraction using two ratios of solvents, plasma:MeOH:CHCl_3_ (1:1:1) and (1:1.5:2.5) ratios and one monophasic extraction with MeOH within 1:8 proportions. All extraction protocols succeeded to provide wide number of metabolites as shown in Figure 1a. Once the chemical redundancy eliminated, the biphasic extraction protocols found 229 compounds all platforms together with 198 metabolites showing a CV <30% in plasma:MeOH:CHCl_3_ (1:1:1) and 230 compounds were detected in plasma:MeOH:CHCl_3_ (1:1.5:2.5) including 184 metabolites showing a CV <30%. The monophasic extraction protocol allows to detect 237 compounds all platforms together with 169 metabolites showing a CV <30%. (see Supplementary Data Table S1). When regarding the number of compounds extracted by only one extraction protocol (Figure 1a), 19 compounds were specific to plasma:MeOH:CHCl_3_ at (1:1:1), nine to plasma: MeOH:CHCl_3_ at (1:1.5:2.5) and 25 were specific to plasma:MeOH (1:8).

Comparing the three preparations, in term of number of metabolite extracted, the plasma:MeOH:CHCl_3_ (1:1:1) preparation was the most suitable preparation.

#### 2.1.2. Milk

The sample used for this step of procedure standardization was a pool of 10 females, with no particular features/traits (including lactation phase, parity, milk production for example). The aim was to get the maximum range of metabolites in the different biofluids, as the subsequent studies for biomarkers research will be carried on several diseases occurring at various stages of lactation.

Highly concentrated in lipids, milk was tested with 3 biphasic preparation protocols (see materials and methods Figure S1b). Once the chemical redundancy eliminated, the milk:H_2_O:MeOH:CHCl_3_ (1:3:4:6) preparations gave the largest number of detected metabolites over 263 compounds were detectable all platforms associated including 221 metabolites having a CV <30% (see Supplementary Data Table S2). When considering the number of specific extracted compounds for each preparation protocol (Figure 1b), milk:H_2_O:MeOH:CHCl_3_ (1:3:4:6) preparation gave the largest specific number of annotated metabolites. In fact this preparation gave 55 specific metabolites compared to 13 unique for milk:MeOH:CHCl_3_ (1:1:1) and four unique for milk:MeOH:CHCl_3_ (1:1.5:2.5).

Comparing the three preparations, in term of number of metabolites extracted and number of unique compounds, the milk:H_2_O:MeOH:CHCl_3_ (1:3:4:6) preparation was the most suitable one.

#### 2.1.3. Feces

Five extraction protocols (see Materials and Methods Figure S1c) were tested on freeze-dried feces. Once the chemical redundancy eliminated, the MeOH:H_2_O (1:1) and (4:1) preparations gave the largest number of detected metabolites over 313 compounds were detectable all platforms associated (see Supplementary Data Table S3). Regarding the number of specific compounds for these two preparations (Figure 1c), the MeOH:H_2_O (4:1) preparation gave the largest specific number of detected metabolites. In fact this preparation gave six specific metabolites compared to two for MeOH:H_2_O (1:1). Comparing the five preparations, in terms of number of metabolite extracted, the MeOH:H_2_O(4:1) preparation was the most suitable preparation.

### 2.2. Matrix Complementarity

**Figure 2:**
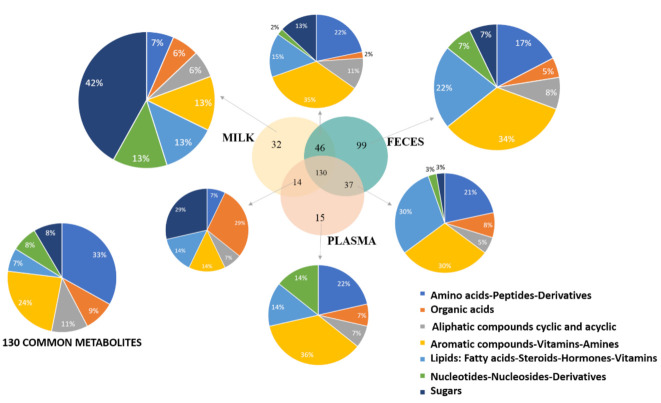
The complementarity of metabolite coverage across the different biological matrices is visualized on a Venn diagram. Pie charts with chemical classes of metabolites found in each Venn section are also displayed.

To assess the complementarity of the three matrices a Venn diagram (Figure 2) was created. This diagram provides an efficient way to visualize the numbers and names (Table S4) of metabolites shared by the different matrices. The metabolites detected were separated into seven different chemical classes. A pie chart was obtained with metabolites found specifically in each Venn section. The seven classes were found in milk with a majority of sugars (42%). These seven categories were also found in feces, but aromatic compounds were predominant in feces (34%). In plasma, the category of sugars was not detected specifically and aromatic compounds and amino acids (respectively 36% and 22%) were the two main classes found.

There were between 14 to 46 common metabolites between two matrices (Table S4) and 130 common metabolites to all three. These 130 common compounds are listed in Table S4. Most of them are amino-acids-peptides derivatives (33%) or aromatic compounds-vitamins-amines (24%).

When comparing with the literature (Table 1), we provided with a confidence level 2 [20] a lot of metabolites implicated in metabolic disease as ketosis or mastitis. Our study highlighted 373 robust molecules describing a healthy individual using 3 matrices (Table S4). These 373 metabolites allow to establish a healthy metabo-atlas as a benchmark to follow goat in herd. Table 1 resumed biomarkers in clinical and subclinical mastisis and in ketosis found in the literature according to the explored matrix. In Table 1, we provided the different matrices where we found the biomarkers described in the literature. As mentioned, a lot of metabolites are present in more than one matrix allowing the health status follow-up simply and easily independently of the biological compartment according to the practice in breeding.

## 3. Discussion

For breeders, it’s very crucial to monitor the health status of their herd but it is sometimes difficult to identify signs of diseases, and therefore in which biological compartment the metabolic modifications happen. The monitoring of the metabolic status using a metabolomic atlas from multimatrices is currently lacking to anticipate and explain a metabolic dysregulation. This study focused on the metabolomics characterization of healthy goats in aim to describe as exhaustively as possible the baseline metabolite coverage in order to create a metabo-atlas. This baseline survey is necessary and very often needed before biomarker studies should be carried on. Three biological matrices were used (milk, plasma and feces) using the complementarity of three analytical platforms, ^1^H-NMR and UHPLC(HILIC)-MS and UHPLC(RP)-MS. Our metabolic coverage (metaboatlas) proposes only metabolites with a CV<30% meaning very reliable compounds found in each healthy sample (*n* = 10) with a validation step of the best operating procedure to prepare them. Moreover, the redundancy of the metabolites in different matrices may provide further information about exchanges between compartments but also to investigate metabolites evolution during pathologies.

Several papers have reported that goat milk is usually prepared with a biphasic protocol [22,23] or with ultrafiltration [16,18]. In our study, testing three biphasic protocol, Milk:H_2_O:MeOH:CHCl_3_ (1:3:4:6) was the most suitable protocol. According to Caboni et al. [22], goat’s and sheep’s milk have a metabolic profile by untargeted GC-MS dependent on the species. This study is very informative in term of milk origin and quality of transformed products but did not provide the metabolic baseline of a normal milk metabolome to follow the animal health. They identified 38 low molecular weight metabolites mainly amino acids, short chain hydroxylated carboxylic acids, organic acids, polyols and sugars. In our study and according to standardized operating procedure we developed, we identified 221 low molecular weight metabolites with 55 exclusively in this biofluid compared to plasma and feces. Plasma and milk shared (130 + 14) 144 metabolites (Table S4). This observation can be explained by the exchange between vascular compartment and glandular tissue. Metabolomics studies usually show the complementarity between milk and plasma to find prognostic biomarker for risk of ketosis or to evaluate the relationship between energy balance and metabolomics profiles in early lactation [9,16]. In milk, for subclinical ketotic conditions, ketone bodies (β-hydroxybutyrate, acetoacetate and acetone) are already accepted as reliable biomarkers [24,25]. In our study, 3-hydroxybutyric acid and acetoacetate are described in milk and plasma and acetone in plasma. Caboni et al. [22] described increased pyruvate and lactate associated with somatic cell count in milk. In our study pyruvate is also present in plasma and lactic acid in the three matrices explored. Xi et al. [8] showed, in an untargeted milk metabolomics study by UPLC-QTOF, biomarkers to differentiate healthy, clinical and subclinical mastitis. As mentioned in Table 1, most of these metabolites are found in feces and/or plasma. Zhang et al. [11] demonstrated that urinary metabolite can highlight ketosis. However, urine is problematic to collect because goat urinary cycle is unpredictable. Monitoring these metabolites can be done through other fluids (like milk, feces or plasma) (Table 1).

According to the literature, plasma can be prepared using ultrafiltration in order to remove macromolecules [9,16] or with a biphasic method for lipid studies [16], only the lipid phase is analyzed. Plasma can be also prepared by a protein precipitation with cold MeOH [26]. In our study, testing two biphasic protocols and one monophasic extraction, plasma:MeOH:CHCl_3_ (1:1:1) was the most suitable protocol with 198 molecules with a CV <30%. Enjalbert et al. [25] showed an increased in the ketone bodies in milk and plasma in true positive cows during subclinical ketosis with a good correlation coefficient between blood and milk for acetone (0.96), acetoacetate (0.74) and BHBA (0.66). This observation confirms that it is possible to follow the health status in different biofluids. Xu et al. [9] showed a relation between energy balance and metabolic profiles in plasma and milk. Some metabolites were found in both plasma and milk but some were found correlated to energy balance only in plasma or in milk. This observation is of importance because it clearly highlights the need to explore several matrices instead of only one matrix.

Sun et al. [26] investigated by GC-TOF/MS the simultaneously responses of four biofluids (serum, milk, rumen fluid and urine) to different forage diets to establish the correlation among biofluids and the forage quality related to milk production and quality in dairy cows. According to the metabolic profiles of the four matrices, authors identified key different metabolic pathways between the two diet groups. Metabolites were identified 165 (rumen fluid), 195 (milk), 218 (serum) and 156 (urine) with 29 common between the four matrices.

In the literature, fecal material could be first freeze-dried and after metabolites could be extracted with EtOH/H_2_O [19] or MeOH followed by a SPE step [27]. In our study, testing one biphasic protocol and four monophasic protocols, feces:H_2_O (4:1) is the most suitable protocol with 313 molecules with a CV <30%. Currently, very few studies have explored fecal metabolites. Scherpenhuizen et al. [19] explored fecal cortisol metabolite concentrations in sheep for noninvasive quantitative analysis of physiological stress in sheep. Dulude-de Broin et al. [27] evaluated faecal metabolites and cortisol in hair as biomarkers of the hypothalamic–pituitary–adrenal -axis activity in captive mountain goats. The cortisol and some glucocorticoid metabolites as corticosterone were found in our study in feces and in milk matrices.

In this publication, the authors deliberately choose to not detail concentration values of each metabolite found in this goat metabo-atlas. Even if it is possible with NMR and usually done, quantification would have been biased by the fact that samples used for this study have been collected in a single farm. It is clearly known that environmental and farm conditions [11], genetic and breed [18], as the most important effects, could hugely impact values of concentration.

Our study makes it possible to cover a wide range of metabolites. A change in this metabolic profile can allow early detection of metabolic dysregulation that can indicate a disease. The next work will be the establishment of correlation between these metabolic dysregulations and diseases. This step will be of a great interest for livestock production, as it will allow a large screening of the metabolism of the animals for an early detection of diseases. Breeders need tools to help them in the daily management of their herd, especially to monitor health status, as some diseases could not show clear clinical signs (subclinical forms) and provoke negative consequences on animal health and products quality.

## 4. Materials and Methods

### 4.1. Sample Collection

For each biofluid/matrix, samples were collected on 10 Alpine goats from a French commercial farm, and pooled, then stored at −80 °C until analysis was performed. Approval by the local ethical committee was not necessary for blood, milk and feces sampling in a commercial farm at the time of the experiment. Samples were collected by staff with skills to conceive and perform experimental procedures from an approved establishment (Allice experimental facilities, n° B-37-175-5), and according to ethical and welfare guidelines usually applied in our experiments.

#### 4.1.1. Plasma

Blood was collected on heparin tubes then centrifuged (15 min, +4 °C, 15,000 g). Pool was formed with plasma supernatant.

#### 4.1.2. Milk

Milk samples were collected in sterilized tubes through manual milking. Pool was constituted with fresh whole milk.

#### 4.1.3. Feces

Fecal samples were collected in sterilized tubes. Samples were individually freeze-dried then pooled and mixed.

### 4.2. Sample Preparation

Sigma Aldrich (Saint-Quentin Fallavier, France) supplied all chemicals.

#### 4.2.1. Plasma

For each extraction protocol, the extraction was repeated 10 times. Three extraction procedures were tested either on 100 uL of plasma, plasma:MeOH:CHCl_3_ (1:1:1) and plasma:MeOH:CHCl_3_ (1:1.5:2.5) or on 200 uL of plasma, plasma:MeOH (1:8). Samples were vortexed after addition of each chemical solvent, kept at −20 °C during 30 min, then centrifuged (10 min, 4 °C, 15,000 g). Supernatant were collected in a glass tube and divided into 2 aliquots (one for the NMR analysis and the other for MS analysis) for further solvent evaporation in a SpeedVac (ThermoFisher, Villebon sur Yvette, France). A graphical visualization of protocols of sample preparation is given in Figure S1a.

#### 4.2.2. Milk

For each extraction protocol, the extraction was repeated 10 times. Three extractions procedures were tested either on 100uL of milk:MeOH:CHCl_3_ (1:1:1) and milk:MeOH:CHCl_3_ (1:1.5:2.5) or 200 uL of milk, milk:H_2_O:MeOH:CHCl_3_ (1:3:4:6). Samples were vortexed after addition of each chemical, kept at −20 °C during 30 min, then centrifuged (10 min, 4 °C, 15,000 g). Supernatant were collected in a glass tube and divided into 2 aliquots (one for the NMR analysis and the other for MS analysis) for further solvent evaporation in a SpeedVac (ThermoFisher). A graphical visualization of protocols of sample preparation is given in Figure S1b.

#### 4.2.3. Feces

Fecal samples were freeze-dried (FreeZone^®^4.5 L, Labconco, Kansan City, MO, USA) at −107 °C, 0.2 mbar for 24 h and then pooled and mixed as previously described by Martias et al. [28].

For each extraction protocol, the extraction was repeated 10 times. Five extraction procedures were tested on 50 mg for each of a pool of dried feces: H_2_O:MeOH:CHCl_3_ (1:1:1), ACN:H_2_O (1:1), MeOH:H_2_O (1:1), MeOH:H_2_O (4:1), MeOH:H_2_O:ACN (1:1:1). Samples were vortexed during 10 min and centrifuged (10 min at 4 °C, 15,000 g). Supernatants were collected in 2 aliquots (one for the NMR analysis and the other for MS analysis) for further solvent evaporation in a SpeedVac (ThermoFisher). A graphical visualization of all sample preparation protocols is given in Figure S1c.

For the MS analysis, dried-residues were dissolved in 150 µL of ACN:H_2_O (4:1). 75 µL were used for HILIC and the remaining phase were evaporated in a Speedvac (ThermoFisher). Then, dried-residues was dissolved in 75µL of MeOH:H_2_O (1:9) for RP-LC. For the ^1^H-NMR analysis, dried-residues were dissolved in 200 μL of a deuterated buffer (0.2 M potassium phosphate buffered deuterium oxide (pH = 7.44 ± 0.5) and 10 μL of deuterium oxide (D_2_O) with external reference [3-trimethylsilylpropionic acid (TSP) at 3.2 mM]).

### 4.3. Data Acquisition

#### 4.3.1. UHPLC-MS

As previously described [29], LC-HRMS analysis was performed on an UPLC Ultimate WPS-3000 system (Dionex, Sunnyvale, CA, USA), coupled to a QExactive-orbitrap mass spectrometer (Thermo Fisher Scientific, San Jose, CA, USA).

The chromatography system was equipped separately with two columns: Reverse Phase Liquid Chromatography (RP-LC) Kinetex XB-C18 (1.7 µm 100 A 150 × 2.1 mm, Phenomenex, Torrance, CA, USA) and a hydrophilic interaction liquid chromatography (HILIC) Cortecs (unbonded silica 1.6 µm 100 A 150 × 2.1 mm, Waters, Dublin, Ireland) as previously described [28].

A head electrospray ionization (HESI) source was used for both chromatography system, operated in positive (ESI+) and negative (ESI−) electrospray ionization modes (one run for each mode), as previously described [28]. Detection was performed with a full-scan acquisition at 70,000 resolution (*m*/*z* = 200) which ranged from 58.0 to 870.0 *m*/*z*, with an automatic gain control (AGC) target of 105 charges and a maximum injection time (IT) of 250 ms as previously described [28]. Xcalibur 2.2 software (Thermo Fisher Scientific, San Jose, CA, USA ) controlled the system.

#### 4.3.2. ^1^H-NMR

As previously described [28], ^1^H-NMR spectra were acquired at 298 K on an AVANCE III HD 600 MHz system (Bruker Biospin, Karlsruhe, Germany). ^1^H-NMR spectra were recorded with «noesypr1d» pulse sequence with a relaxation delay of 20 s, on a spectral width of 12 ppm, a time domain of 64 k points, an acquisition time of 4.55 s, with 64 FIDs and 8 dummy scans.

### 4.4. Data Processing

#### 4.4.1. UHPLC-MS

Data processing was carried out with Xcalibur 2.2 software (Thermo Fisher Scientific, San Jose, CA, USA) as previously described [30]. A library of 495 standard metabolites (Mass Spectroscopy Metabolite Library of Standards MSMLS^®^ (IROA Technologies™), Sea Girt, NJ, USA) was analyzed with the same gradient of mobile phases and in the same conditions. The 495 molecules were divided into 42 pools of 12 molecules dispatched on a 96-well plate. The pools were made with molecules having different masses. Each pool is injected in full-scan acquisition mode then injected a second time in MS^2^ (PRM –parallel reaction monitoring- mode). For each metabolite, the high-resolution mass and retention time parameters have been integrated into the “Thermo Xcalibur processing setup” (ThermoFisher Scientific) which is an automatic integration software. Once this list of metabolites has been produced, it is used to find and integrate each of these metabolites in the samples with a precision on the mass of 5 ppm (Mode +) and 10 ppm (Mode −) around the theoretical mass. A window of 12 s relative to the retention time observed for the standards was used in order to select the metabolites to be integrated.

All samples were then processed with this integration method to create a result file using “Thermo Xcalibur Quan browser”. All the integrations created in this way were checked and then exported to an .xls file containing the areas of each metabolite. Each peak area was normalized to the total of peak areas of interest.

#### 4.4.2. ^1^H-NMR

As previously described [28], spectra were processed using TopSpin 3.6 software (Bruker Biospin GmbH, Rheinstetten, Germany) and were reduced to buckets of variable width using AMIX software (Analysis of MIXture, version 3.2, Bruker Biospin GmbH, Rheinstetten, Germany). Bucket identification was done using ChenomX software (NMR Suite 7.7, ChenomX version 8.6, Edmonton, Canada) and literature [31]

### 4.5. Data Fusion

Once the pre-processing of the spectral data was done, Xcalibur and ChenomX generated respectively a compound list for LC-MS data and for NMR data. Generated names by ChenomX and IROA^®^ database are loaded in metaboanalyst (https://www.metaboanalyst.ca/ accessed on 10 of June 2021) in order to have a generic name for all platforms. Thanks to this homogeneity of names, KEGG and HMDB numbers were attributed for each list of metabolites coming from the different platforms. Thus, NMR list, RP-MS list and HILIC-MS list could be merged. When metabolites were detected by several platforms, redundant metabolites (generic name based on their KEGG, HMDB or PubChem numbers) were deleted based on their signal variation coefficient (CV) comparing the different platforms. To eliminate the redundancy, the metabolites with the largest CV was deleted and the information coming from the platform having the most reproducible signal was kept.

### 4.6. Data Analysis

The data generated from each matrix, for each extraction protocol and from each platform were gathered in an .xls file with generic name obtained with metaboanalyst (https://www.metaboanalyst.ca/ accessed on 10 June 2021 ) as mentioned in data fusion. The number of metabolites, unique metabolite and each coefficient of variation (CV%) were calculated in order to validate the reproducibility of the workflow and to choose the best extraction protocol.

## 5. Conclusions

This study presents the first metabolomics fingerprinting strategy for multimatrices (plasma, milk and feces) analysis using a multiplatform approach ((NMR (^1^H), RP and HILIC-MS) on goats. We validate a unique preparation by biological matrix to be exploitable by NMR and LC-MS. This approach makes it possible to see the complementarity between the matrices and to consider the permeation of metabolites across biological compartments. It will therefore allow a broad description of the goat metabolome. Thanks to the metabolic monitoring of a healthy individual, we will be able to demonstrate a dysregulation the goat’s health status at an early stage.

## Figures and Tables

**Table 1:** Metabolites of interest differentially present in different matrices according to the literature and found in one, two or the three matrices exploited in our study with a CV <30% according to the best preparation procedure. (↗ increased, ↘ decreased, NF = not found).

Disease/Status	Mastitis Xi et al. (2017) [8]			Ketosis Zhang G. et al. 2021 [11]		Mastitis Sundekilde UK et al. 2013 [21]	Healthy Martias C. et al. The Present Study
	Milk Cow			Urinary Cow	Urinary Cow	Milk Cow	Plasma, Milk and Feces Goat
	Significantly Different Between			Metabolite Alteration		Strong Association With Stomatic Cell Count (SCC)	Matrices Where Metabolites Are Present
	Healthy and Clinical	Healthy and Subclinical	Subclinical and Clinical	Preceding Ketosis	During Ketosis		
3-Hydroxy-butyric acid					↗	↗	Milk, Plasma
4-Hydroxy-phenyl-lactate	↘		↘				Feces, Plasma
4-Hydroxy-phenyl-pyruvate	↘						Feces
5-hydroxy-L-tryptophane		↘					Feces
Acetic acid						↗	Milk, Feces, Plasma
Acetoacetic acid					↗		Milk, Plasma
L-Arginine	↗	↗					Milk, Feces, Plasma
Ascorbic acid				↗	↗		NF
Benzoic acid		↘					Feces
Butyrate						↗	Feces, Plasma
L-Carnitine	↘	↘					Milk, Feces, Plasma
Citrate	↘						Milk, Plasma
Dimethylglycine		↘					Plasma
D-Lactose			↘			↘	Milk, Plasma
Dopamine	↗						Feces
Fumarate						↘	Milk, feces
Glucose	↘						Milk, Feces, Plasma
Glucose-1-phosphate	↘	↘	↘				Milk
Guanosine monophosphate	↘						Plasma
Hippurate	↘					↘	Milk, Feces, Plasma
Isocitric acid				↗			Feces, Plasma
L-Isoleucine	↗					↗	Milk, Feces, Plasma
L-Lactic acid					↗	↗	Milk, Feces, Plasma
Malate	↘						Milk, Feces, Plasma
Oxoglutarate			↘				Milk
Phosphocholine	↘		↘				Milk, Feces, Plasma
L-Proline	↗						Milk, Feces, Plasma
Riboflavin			↘				Milk, Feces
sn-Glycero-3-phosphocholine	↘						Milk, Feces, Plasma
Uridine	↘						Milk, Feces, Plasma
L-Valine	↗						Milk, Feces, Plasma

## Data Availability

Data are contained on the supplementary material.

## Supplementary Materials

The following are available online at https://www.mdpi.com/article/10.3390/metabo11100681/s1. Figure S1: Schematic presentation of the preparations procedures applied on the pool of plasma (a), milk (b) and feces (c) for the selection of the optimum protocol for the maximum metabolome coverage by UHPLC-MS and 1H-NMR., Table S1: Number of extracted metabolites and coefficient of validation in blood for the different extraction methods using 1H-NMR and UHPLC-MS, Table S2: Number of extracted metabolites and coefficient of validation in milk for the different extraction methods using 1H-NMR and UHPLC-MS, Table S3: Number of extracted metabolites and coefficient of validation in fecal for the different extraction methods using 1H-NMR and UHPLC-MS, Table S4: Metabolites extracted from each biological matrix according to the best preparation procedure, with a CV <30%, Table S5: Metabolites detected by platform in plasma, milk and feces. In bold the platform where the metabolite was annotated with the lowest CV (best reproducibility).
