# Supplementary material for: Analytical Methodology for a Metabolome Atlas of Goat’s Plasma, Milk and Feces Using 1H-NMR and UHPLC-HRMS
Source: Metabolites. 2021 Oct 4;11(10):681. doi: 10.3390/metabo11100681 (PMC8537934; doi:10.3390/metabo11100681)
Supplement: Supplementary file 1 [file metabolites-11-00681-s001.zip › metabolites-1382498-supplementary.pdf]

SUPPLEMENTARY DATA

Analytical methodology for a metabolome atlas of goat's plasma, milk and feces using 1H-NMR and UHPLC-HRMS

Cécile Martias[1], Julie Gatien[2], Lea Roch[2], Nadine Baroukh[1], Sylvie Mavel[1], Antoine Lefevre[1], Fréderic Montigny[1], Laurent Schibler[2], Patrick Emond[1,3] ,Lydie Nadal-Desbarats[1]

1 - UMR 1253, iBrain, University of Tours, Inserm, Tours, France
2 – ALLICE, Phenotyping station, Nouzilly, France
3-CHRU Tours, Biochemistry and molecular biology department, Tours, France

SAMPLE PREPARATION

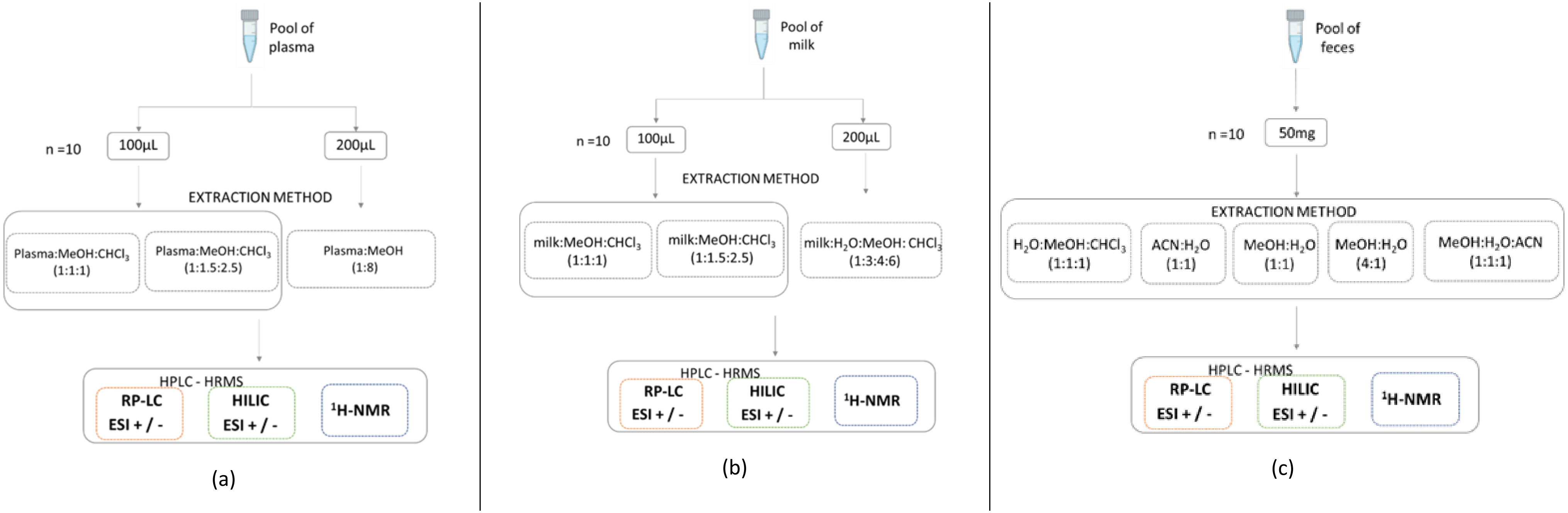

Figure S1. Schematic presentation of the preparation procedures applied on the pool of plasma (a), milk (b) and feces (c) for the selection of the optimum protocol for the maximum metabolome coverage by UHPLC-MS and ^{1}H-NMR.

PLASMA

		RP-LC	HILIC	NMR	Total
Plasma:MeOH :$CHCl_3$ (1 :1 :1) (n=10)	Number of metabolites :	137	131	39	229
	CV<30%	108	97	39	198
	CV<20%	81	73	39	161
	CV<10%	32	31	29	78
	CV % mean (min-max)	22% (4%-93%)	27% (3%-135%)	8% (4%-15%)	18% (3%-93%)
Plasma:MeOH :$CHCl_3$ (1 :2.5 :1) (n=10)	Number of metabolites :	145	129	36	230
	CV<30%	123	84	36	184
	CV<20%	103	65	35	160
	CV<10%	40	21	24	69
	CV % mean (min-max)	18% (4%-92%)	26% (3%-135%)	8% (3%-20%)	19% (3%-92%)
Plasma:MeOH (1 :8) (n=10)	Number of metabolites :	136	153	38	237
	CV<30%	120	61	38	169
	CV<20%	98	33	38	140
	CV<10%	41	15	30	78
	CV % mean (min-max)	16% (1%-50%)	41% (2%-120%)	6% (1%-19%)	24% (1%-120%)

Table S1: Number of extracted metabolites and coefficient of validation in blood for the different extraction methods using ^{1}H-NMR and UHPLC-MS

MILK

		RP-LC	HILIC	NMR	Total
Milk:MeOH :$CHCl_3$ (1 :1 :1) (n=10)	Number of metabolites :	142	131	35	234
	CV<30%	126	97	35	200
	CV<20%	111	58	35	166
	CV<10%	68	13	28	95
	CV % mean (min-max)	14% (1%-102%)	25% (1%-107%)	7% (3%-17%)	17% (1%-84%)
Milk:MeOH :$CHCl_3$ (1 :2.5 :2.5) (n=10)	Number of metabolites :	144	131	35	237
	CV<30%	95	90	35	172
	CV<20%	69	49	34	125
	CV<10%	19	8	23	45
	CV % mean (min-max)	30% (4%-175%)	27% (1%-110%)	9% (7%-20%)	25% (1%-122%)
Milk:H_2O:MeOH :$CHCl_3$ (1 :3 :4 :6) (n=10)	Number of metabolites :	139	151	33	263
	CV<30%	121	109	33	221
	CV<20%	107	107	32	187
	CV<10%	65	35	21	100
	CV % mean (min-max)	15% (2%-78%)	20% (2%-84%)	8% (2%-22%)	17% (2%-84%)

Table S2: Number of extracted metabolites and coefficient of validation in milk for the different extraction methods using ^{1}H-NMR and UHPLC-MS

FECES

		RP-LC	HILIC	NMR	Total
H_2O:MeOH :$CHCl_3$ (1 :1 :1) (n=10)	Number of metabolites :	236	250	28	347
	CV<30%	194	199	21	286
	CV<20%	175	143	17	241
	CV<10%	141	109	7	192
	CV % mean (min-max)	15% (2%-92%)	21% (3%-168%)	21% (5%-53%)	17% (2%-168%)
ACN:H_2O 1 :1 (n=10)	Number of metabolites :	236	256	28	348
	CV<30%	195	220	28	306
	CV<20%	169	196	28	280
	CV<10%	111	124	8	186
	CV % mean (min-max)	17% (2%-103%)	17% (3%-149%)	11% (6%-17%)	14% (2%-97%)
MeOH:H_2O 1 :1 (n=10)	Number of metabolites :	238	262	28	355
	CV<30%	198	222	28	313
	CV<20%	187	191	26	289
	CV<10%	137	132	13	215
	CV % mean (min-max)	15% (2%-98%)	16% (2%-157%)	11% (5%-24%)	14% (2%-157%)
MeOH:H_2O 4:1 (n=10)	Number of metabolites :	240	261	28	353
	CV<30%	201	221	25	313
	CV<20%	188	204	22	291
	CV<10%	138	140	19	222
	CV % mean (min-max)	15% (2%-99%)	16% (3%-119%)	13% (4%-54%)	13% (2%-119%)
MeOH:H_2O:ACN 1:1: (n=10)	Number of metabolites :	236	254	28	348
	CV<30%	201	219	28	310
	CV<20%	185	203	28	296
	CV<10%	149	138	16	225
	CV % mean (min-max)	14% (2%-95%)	15% (1%-119%)	9% (5%-15%)	13% (1%-119%)

Table S3: Number of extracted metabolites and coefficient of validation in fecal for the different extraction methods using ^{1}H-NMR and UHPLC-MS

List of metabolites found in each biological matrix or shared between two or three matrices

Table S4: Metabolites extracted from each biological matrix according to the best preparation procedure, with a CV<30%.

Only in plasma				
**Metabolite**	**Chemical class**	**HMDB**	**PubChem**	**KEGG**
**Dihydroxyacetone phosphate**	Acides phosphoriques	HMDB0001473	668	C00111
**Acetone**	Aliphatic compounds cyclic and acyclic	HMDB0001659	180	C00207
**Dimethylglycine**	Amino acids-Peptides-Derivatives	HMDB0000092	673	C01026
**L-Cystine**	Amino acids-Peptides-Derivatives	HMDB0000192	67678	C00491
**Thyroxine**	Amino acids-Peptides-Derivatives	HMDB0000248	5819	C01829
**1-Hydroxy-2-naphthoate**	Aromatic compounds-Vitamins-Amines	METPA0374		C03203
**4-Hydroxycinnamic acid**	Aromatic compounds-Vitamins-Amines	HMDB0002035	637542	C00811
**Bilirubin**	Aromatic compounds-Vitamins-Amines	HMDB0000054	21252250	C00486
**Phenylacetic acid**	Aromatic compounds-Vitamins-Amines	HMDB0000209	999	C07086
**Phenylpyruvic acid**	Aromatic compounds-Vitamins-Amines	HMDB0000205	997	C00166
**(S)-3-Hydroxyisobutyric acid**	Lipids: Fatty acids-Steroids-Hormones-Vitamins	HMDB0000023	87	C06001
**Methyl jasmonate**	Lipids: Fatty acids-Steroids-Hormones-Vitamins	HMDB0036583	5367719	C11512
**Guanosine monophosphate**	Nucleotides-Nucleosides-Derivatives	HMDB0001397	6804	C00144
**Xanthosine**	Nucleotides-Nucleosides-Derivatives	HMDB0000299	64959	C01762
**Pyruvic acid**	Organic acids	HMDB0000243	1060	C00022

Only in Milk				
**Metabolite**	**Chemical class**	**HMDB**	**PubChem**	**KEGG**
**4-Methylcatechol**	Aromatic compounds-Vitamins-Amines	HMDB0000873	9958	C06730
**Adenosine monophosphate**	Nucleotides-Nucleosides-Derivatives	HMDB0000045	6083	C00020
**ADP-glucose**	Nucleotides-Nucleosides-Derivatives	HMDB0006557	16500	C00498
**Butanal**	Aliphatic compounds cyclic and acyclic	HMDB0003543	261	C01412
**Cellobiose**	Sugars	HMDB00055	10712	C00185
**D-Fructose**	Sugars	HMDB00660	5984	C00095
**D-Tagatose**	Sugars	HMDB03418	439312	C00795
**D-Xylitol**	Sugars	HMDB02917	6912	C00379
**Dethiobiotin**	Lipids: Fatty acids-Steroids-Hormones-Vitamins	HMDB0003581	445027	C01909
**Dimethyl sulfone**	Organic acids	HMDB0004983	6213	C11142
**Galactaric acid**	Sugars	HMDB0000639	3037582	C00879
**Galactose 1-phosphate**	Sugars	HMDB0000645	123912	C00446
**Glucaric acid**	Sugars	HMDB0000663	33037	C00818
**Glycerol 3-phosphate**	Lipids: Fatty acids-Steroids-Hormones-Vitamins	HMDB0000126	439162	C00093
**Heptanoic acid**	Lipids: Fatty acids-Steroids-Hormones-Vitamins	HMDB0000666	8094	C17714
**Hypotaurine**	Organic acids	HMDB0000965	107812	C00519
**L-Fucose**	Sugars	HMDB0000174	3034656	C01019
**L(-)-Nicotine**	Aromatic compounds-Vitamins-Amines	HMDB0001934	89594	C00745
**Melatonin**	Aromatic compounds-Vitamins-Amines	HMDB0001389	896	C01598
**N-Methylhydantoin**	Aliphatic compounds cyclic and acyclic	HMDB03646	69217	C02565

**Oxoglutaric acid**	Lipids: Fatty acids-Steroids-Hormones-Vitamins	HMDB0000208	51	C00026
**Palatinose**	Sugars	HMDB0256083	52994241	C01742
**Phenylacetylglycine**	Amino acids-Peptides-Derivatives	HMDB00821	68144	C05598
**Phosphocreatine**	Amino acids-Peptides-Derivatives	HMDB0001511	587	C02305
**Phosphocreatine**	Amino acids-Peptides-Derivatives	HMDB0001511	587	C02305
**Sorbitol**	Sugars	HMDB00247	5780	C00794
**Sucrose**	Sugars	HMDB0000258	5988	C00089
**Theophylline**	Aromatic compounds-Vitamins-Amines	HMDB0001889	2153	C07130
**Uridine-5-diphospho-n-acetylglucosamine**	Nucleotides-Nucleosides-Derivatives	HMDB00290	445675	C00043
**Uridine diphosphate-N-acetylgalactosamine**	Nucleotides-Nucleosides-Derivatives	HMDB0000304	1167	C00203
**Uridine diphosphate glucose**	Sugars	HMDB0000286	53477679	C00029
**Uridine diphosphategalactose**	Sugars	HMDB0000302	18068	C00052

Milk Plasma				
**Metabolite**	**Chemical class**	**HMDB**	**PubChem**	**KEGG**
**Glycolaldehyde dimer**	Aliphatic compounds cyclic and acyclic		186078	
**Gamma-Aminobutyric acid**	Amino acids-Peptides-Derivatives	HMDB0000112	223130	C00334
**Dimethylamine**	Aromatic compounds-Vitamins-Amines	HMDB0000087	674	C00543
**Theobromine**	Aromatic compounds-Vitamins-Amines	HMDB0002825	5429	C07480
**3-Hydroxybutyric acid**	Lipids: Fatty acids-Steroids-Hormones-Vitamins	HMDB0000357	441	C01089
**Lithocholyltaurine**	Lipids: Fatty acids-Steroids-Hormones-Vitamins	HMDB0000722	53477716	C02592
**3-Hydroxymethylglutaric acid**	Organic acids	HMDB0000355	1662	C03761
**Acetoacetic acid**	Organic acids	HMDB0000060	96	C00164
**Citric acid**	Organic acids	HMDB0000094	311	C00158
**Methylmalonic acid**	Organic acids	HMDB0000202	487	C02170
**Allose**	Sugars	HMDB0001151	12285879	C01487
**Alpha-Lactose**	Sugars	HMDB0000186	84571	C00243
**D-Galactose**	Sugars	HMDB0000143	439357	C00984
**L-Sorbose**	Sugars	HMDB0001266	441484	C08356

Milk Feces				
**Metabolite**	**Chemical class**	**HMDB**	**PubChem**	**KEGG**
**1-Aminocyclopropanecarboxylic acid**	Amino acids-Peptides-Derivatives	HMDB0036458	535	C01234
**2-Hydroxypyridine**	Aromatic compounds-Vitamins-Amines	HMDB0013751	8871	C02502
**3-Aminoisobutanoic acid**	Amino acids-Peptides-Derivatives	HMDB0003911	64956	C05145
**3-Methyladenine**	Aromatic compounds-Vitamins-Amines	HMDB0011600	1673	C00913
**4-Hydroxybenzaldehyde**	Aromatic compounds-Vitamins-Amines	HMDB0011718	126	C00633
**5-Hydroxylysine**	Amino acids-Peptides-Derivatives	HMDB0000450	4433	C16741
**Acetamide**	Aromatic compounds-Vitamins-Amines	HMDB0031645	178	C06244
**Anserine**	Amino acids-Peptides-Derivatives	HMDB0000194	112072	C01262
**Cholic acid**	Lipids: Fatty acids-Steroids-Hormones-Vitamins	HMDB0000619	221493	C00695
**Citraconic acid**	Lipids: Fatty acids-Steroids-Hormones-Vitamins	HMDB0000634	643798	C02226
**Corticosterone**	Lipids: Fatty acids-Steroids-Hormones-Vitamins	HMDB0001547	5753	C02140
**Cortisol**	Lipids: Fatty acids-Steroids-Hormones-Vitamins	HMDB0000063	657311	C00735
**Cortisol-21-acetate**	Lipids: Fatty acids-Steroids-Hormones-Vitamins		5744	C02821
**Cyclic AMP**	Nucleotides-Nucleosides-Derivatives	HMDB0000058	6076	C00575
**Diacetyl**	Aliphatic compounds cyclic and acyclic	HMDB0003407	650	C00741

**Epinephrine**	Aromatic compounds-Vitamins-Amines	HMDB0000068	5816	C00788
**Ethanolamine**	Aliphatic compounds cyclic and acyclic	HMDB0000149	700	C00189
**Fumaric acid**	Lipids: Fatty acids-Steroids-Hormones-Vitamins	HMDB0000134	444972	C00122
**Glucosamine 6-phosphate**	Sugars	HMDB0001254	439217	C00352
**Glucose 6-phosphate**	Sugars	HMDB0001401	5958	C00092
**Glyceraldehyde-3-phosphatediethylacetal**	Aliphatic compounds cyclic and acyclic		3334922	
**Imidazoleacetic acid**	Aromatic compounds-Vitamins-Amines	HMDB0002024	96215	C02835
**Indolepyruvate**	Aromatic compounds-Vitamins-Amines	HMDB0060484	803	C00331
**Kynurenic acid**	Amino acids-Peptides-Derivatives	HMDB0000715	3845	C01717
**L-Acetylcarnitine**	Lipids: Fatty acids-Steroids-Hormones-Vitamins	HMDB0000201	7045767	C02571
**L-Allothreonine**	Amino acids-Peptides-Derivatives	HMDB0004041	99289	C05519
**L-Dihydroorotic acid**	Aliphatic compounds cyclic and acyclic	HMDB0003349	439216	C00337
**Mannitol**	Sugars	HMDB0000765	6251	C00392
**N-Acetyl-D-glucosamine**	Sugars	HMDB0000215	439174	C00140
**N-Acetylgalactosamine**	Sugars	HMDB0000212	84265	C01074
**N-Acetyl-L-methionine**	Amino acids-Peptides-Derivatives	HMDB0011745	6180	C02712
**N-Acetylmannosamine**	Sugars	HMDB0001129	11096158	C00645
**Niacinamide**	Aromatic compounds-Vitamins-Amines	HMDB0001406	936	C00153
**Nicotinic acid**	Aromatic compounds-Vitamins-Amines	HMDB0001488	938	C00253

**N-Methyltryptamine**	Aromatic compounds-Vitamins-Amines	HMDB0004370	6088	C06213
**N-Trimethyllysine**	Amino acids-Peptides-Derivatives		440121	C03793
**Oxfenicine**	Amino acids-Peptides-Derivatives	HMDB0246152	36143	D05292
**Pantolactone**	Aliphatic compounds cyclic and acyclic	HMDB0059876	439368	C01012
**Pterin**	Aromatic compounds-Vitamins-Amines	HMDB0000802	73000	C00715
**Pyrrole-2-carboxylic acid**	Amino acids-Peptides-Derivatives	HMDB0004230	12473	C05942
**Riboflavin**	Aromatic compounds-Vitamins-Amines	HMDB0000244	493570	C00255
**Salicyluric acid**	Aromatic compounds-Vitamins-Amines	HMDB0000840	10253	C07588
**Thymine**	Aromatic compounds-Vitamins-Amines	HMDB0000262	1135	C00178
**trans-Aconitic acid**	Organic acids	HMDB0000958	444212	C02341
**trans-Ferulic acid**	Aromatic compounds-Vitamins-Amines	HMDB0000954	445858	C01494
**Tryptophanol**	Aromatic compounds-Vitamins-Amines	HMDB0003447	10685	C00955

**Milk Feces Plasma**				
**Metabolite**	Chemical class	HMDB	PubChem	KEGG
**Acetylcholine**	Aliphatic compounds cyclic and acyclic	HMDB0000895	6060	C01996
**Allantoin**	Aliphatic compounds cyclic and acyclic	HMDB0000462	204	C01551
**Biotin**	Aliphatic compounds cyclic and acyclic	HMDB0000030	171548	C00120
**Choline**	Aliphatic compounds cyclic and acyclic	HMDB0000097	305	C00114
**Creatinine**	Aliphatic compounds cyclic and acyclic	HMDB0000562	588	C00791
**Cyclohexane-1,2-Diol**	Aliphatic compounds cyclic and acyclic		13601	C03739
**L-Carnitine**	Aliphatic compounds cyclic and acyclic	HMDB0000062	2724480	C00318
**Methylguanidine**	Aliphatic compounds cyclic and acyclic	HMDB0001522	10111	C02294
**O-Phosphoethanolamine**	Aliphatic compounds cyclic and acyclic	HMDB0000224	1015	C00346
**Pantothenic acid**	Aliphatic compounds cyclic and acyclic	HMDB0000210	6613	C00864
**Phosphorylcholine**	Aliphatic compounds cyclic and acyclic	HMDB0001565	8691	C00588
**Propanal**	Aliphatic compounds cyclic and acyclic	HMDB0003366	527	C00479
**Trimethylamine**	Aliphatic compounds cyclic and acyclic	HMDB0000906	1146	C00565
**Ureidopropionic acid**	Aliphatic compounds cyclic and acyclic	HMDB0000026	111	C02642
**2,4-Dihydroxypyrimidine-5-carboxylic acid**	Amino acids-Peptides-Derivatives		90301	C03030
**3-Methylhistidine**	Amino acids-Peptides-Derivatives	HMDB0000479	64969	C01152
**4-Hydroxy-L-proline**	Amino acids-Peptides-Derivatives	HMDB0006055	69248	C01015
**5-Aminolevulinic acid**	Amino acids-Peptides-Derivatives	HMDB0001149	137	C00430

**Acetylglycine**	Amino acids-Peptides-Derivatives	HMDB0000532	10972	
**Beta-Alanine**	Amino acids-Peptides-Derivatives	HMDB0000056	239	C00099
**Betaine**	Amino acids-Peptides-Derivatives	HMDB0000043	247	C00719
**Citrulline**	Amino acids-Peptides-Derivatives	HMDB0000904	9750	C00327
**Creatine**	Amino acids-Peptides-Derivatives	HMDB0000064	586	C00300
**Formyl-L-methionylpeptide**	Amino acids-Peptides-Derivatives		13611	C11439
**Glycine**	Amino acids-Peptides-Derivatives	HMDB0000123	750	C00037
**Guanidoacetic acid**	Amino acids-Peptides-Derivatives	HMDB0000128	763	C00581
**Hippuric acid**	Amino acids-Peptides-Derivatives	HMDB0000714	464	C01586
**L-Arginine**	Amino acids-Peptides-Derivatives	HMDB0000517	6322	C00062
**L-Asparagine**	Amino acids-Peptides-Derivatives	HMDB0000168	6267	C00152
**L-Aspartic acid**	Amino acids-Peptides-Derivatives	HMDB0000191	5960	C00049
**L-Glutamic acid**	Amino acids-Peptides-Derivatives	HMDB0000148	33032	C00025
**L-Glutamine**	Amino acids-Peptides-Derivatives	HMDB0000641	5961	C00064
**L-Histidine**	Amino acids-Peptides-Derivatives	HMDB0000177	6274	C00135
**L-Homoserine**	Amino acids-Peptides-Derivatives	HMDB0000719	12647	C00263
**L-Isoleucine**	Amino acids-Peptides-Derivatives	HMDB0000172	6306	C00407
**L-Kynurenine**	Amino acids-Peptides-Derivatives	HMDB0000684	161166	C00328
**L-Leucine**	Amino acids-Peptides-Derivatives	HMDB0000687	6106	C00123
**L-Lysine**	Amino acids-Peptides-Derivatives	HMDB0000182	5962	C00047
**L-Methionine**	Amino acids-Peptides-Derivatives	HMDB0000696	6137	C00073

**L-Norleucine**	Amino acids-Peptides-Derivatives	HMDB0001645	21236	C01933
**L-Phenylalanine**	Amino acids-Peptides-Derivatives	HMDB0000159	6140	C00079
**L-Proline**	Amino acids-Peptides-Derivatives	HMDB0000162	145742	C00148
**L-Serine**	Amino acids-Peptides-Derivatives	HMDB0000187	5951	C00065
**L-Threonine**	Amino acids-Peptides-Derivatives	HMDB0000167	6288	C00188
**L-Tryptophan**	Amino acids-Peptides-Derivatives	HMDB0000929	6305	C00078
**L-Tyrosine**	Amino acids-Peptides-Derivatives	HMDB0000158	6057	C00082
**L-Valine**	Amino acids-Peptides-Derivatives	HMDB0000883	6287	C00183
**N-Acetyl-L-alanine**	Amino acids-Peptides-Derivatives	HMDB0000766	88064	
**N-Acetyl-L-phenylalanine**	Amino acids-Peptides-Derivatives	HMDB0000512	74839	C03519
**N-Acetylleucine**	Amino acids-Peptides-Derivatives	HMDB0011756	70912	C02710
**N-Acetylserine**	Amino acids-Peptides-Derivatives	HMDB0002931	65249	
**N-acetyltryptophan**	Amino acids-Peptides-Derivatives	HMDB0013713	700653	
**Ornithine**	Amino acids-Peptides-Derivatives	HMDB0000214	6262	C00077
**Orotic acid**	Amino acids-Peptides-Derivatives	HMDB0000226	967	C00295
**Pipecolic acid**	Amino acids-Peptides-Derivatives	HMDB0000070	849	C00408
**Pyroglutamic acid**	Amino acids-Peptides-Derivatives	HMDB0000267	7405	C01879
**Sarcosine**	Amino acids-Peptides-Derivatives	HMDB0000271	1088	C00213
**2-Aminophenol**	Aromatic compounds-Vitamins-Amines	METPA0236		C01987
**3-(2-Hydroxyphenyl)propanoic acid**	Aromatic compounds-Vitamins-Amines	HMDB0033752	873	C01198
**3-Hydroxyphenylacetic acid**	Aromatic compounds-Vitamins-Amines	HMDB0000440	12122	C05593

**3-Methyldioxyindole**	Aromatic compounds-Vitamins-Amines	HMDB0004186	151066	C05834
**3,4-Dihydroxybenzeneacetic acid**	Aromatic compounds-Vitamins-Amines	HMDB0001336	547	C01161
**5-Hydroxyindoleacetic acid**	Aromatic compounds-Vitamins-Amines	HMDB0000763	1826	C05635
**Adenine**	Aromatic compounds-Vitamins-Amines	HMDB0000034	190	C00147
**Benzyl alcohol**	Aromatic compounds-Vitamins-Amines	HMDB0003119	244	C03485
**Cytosine**	Aromatic compounds-Vitamins-Amines	HMDB0000630	597	C00380
**Gentisic acid**	Aromatic compounds-Vitamins-Amines	HMDB0000152	3469	C00628
**Guanine**	Aromatic compounds-Vitamins-Amines	HMDB0000132	764	C00242
**Homogentisic acid**	Aromatic compounds-Vitamins-Amines	HMDB0000130	780	C00544
**Homovanillic acid**	Aromatic compounds-Vitamins-Amines	HMDB0000118	1738	C05582
**Hypoxanthine**	Aromatic compounds-Vitamins-Amines	HMDB0000157	790	C00262
**Indoleacetic acid**	Aromatic compounds-Vitamins-Amines	HMDB0000197	802	C00954
**Indoxyl sulfate**	Aromatic compounds-Vitamins-Amines	HMDB0000682	10258	
**Lumichrome**	Aromatic compounds-Vitamins-Amines	METPA0216		C01727
**N-Acetylserotonin**	Aromatic compounds-Vitamins-Amines	HMDB0001238	903	C00978
**N,N-Dimethylphenethylamine**	Aromatic compounds-Vitamins-Amines	HMDB0032243	86536	
**Ortho-Hydroxyphenylacetic acid**	Aromatic compounds-Vitamins-Amines	HMDB0000669	11970	C05852
**p-Hydroxyphenylacetic acid**	Aromatic compounds-Vitamins-Amines	HMDB0000020	127	C00642
**Paraxanthine**	Aromatic compounds-Vitamins-Amines	HMDB0001860	4687	C13747
**Protocatechuic acid**	Aromatic compounds-Vitamins-Amines	HMDB0001856	72	C00230
**Pyrocatechol**	Aromatic compounds-Vitamins-Amines	HMDB0000957	3390	C15571

**trans-Cinnamic acid**	Aromatic compounds-Vitamins-Amines	HMDB0000930	444539	C10438
**Trigonelline**	Aromatic compounds-Vitamins-Amines	HMDB0000875	5570	C01004
**Uracil**	Aromatic compounds-Vitamins-Amines	HMDB0000300	1174	C00106
**Uric acid**	Aromatic compounds-Vitamins-Amines	HMDB0000289	1175	C00366
**Urocanic acid**	Aromatic compounds-Vitamins-Amines	HMDB0000301	736715	C00785
**Xanthine**	Aromatic compounds-Vitamins-Amines	HMDB0000292	1188	C00385
**Thymine**	Aromatic compounds-Vitamins-Amines	HMDB0000262	1135	C00178
**12-Hydroxydodecanoic acid**	Lipids: Fatty acids-Steroids-Hormones-Vitamins	HMDB0002059	79034	C08317
**2-Hydroxybutyric acid**	Lipids: Fatty acids-Steroids-Hormones-Vitamins	HMDB0000008	11266	C05984
**4-Trimethylammoniobutanoic acid**	Lipids: Fatty acids-Steroids-Hormones-Vitamins	HMDB0001161	134	C01181
**Caproic acid**	Lipids: Fatty acids-Steroids-Hormones-Vitamins	HMDB0000535	8892	C01585
**Ethylmalonic acid**	Lipids: Fatty acids-Steroids-Hormones-Vitamins	HMDB0000622	11756	
**Galactitol**	Lipids: Fatty acids-Steroids-Hormones-Vitamins	HMDB0000107	11850	C01697
**Glycerophosphocholine**	Lipids: Fatty acids-Steroids-Hormones-Vitamins	HMDB0000086	71920	C00670
**Glycocholic acid**	Lipids: Fatty acids-Steroids-Hormones-Vitamins	HMDB0000138	23617285	C01921
**Ketoleucine**	Lipids: Fatty acids-Steroids-Hormones-	HMDB0000695	70	C00233

	Vitamins			
**1-Methyladenosine**	Nucleotides-Nucleosides-Derivatives	HMDB0003331	27476	C02494
**5'-Methylthioadenosine**	Nucleotides-Nucleosides-Derivatives	HMDB0001173	439176	C00170
**Adenosine**	Nucleotides-Nucleosides-Derivatives	HMDB0000050	60961	C00212
**Deoxycytidine**	Nucleotides-Nucleosides-Derivatives	HMDB0000014	13711	C00881
**Deoxyguanosine**	Nucleotides-Nucleosides-Derivatives	HMDB0000085	187790	C00330
**Guanosine**	Nucleotides-Nucleosides-Derivatives	HMDB0000133	6802	C00387
**Inosine**	Nucleotides-Nucleosides-Derivatives	HMDB0000195	6021	C00294
**N6-(delta2-Isopentenyl)-adenine**	Nucleotides-Nucleosides-Derivatives	METPA1684		C04083
**Thymidine**	Nucleotides-Nucleosides-Derivatives	HMDB0000273	5789	C00214
**Uridine**	Nucleotides-Nucleosides-Derivatives	HMDB0000296	6029	C00299
**3-Hydroxycapric acid**	Organic acids	HMDB0002203	26612	
**4-Acetamidobutanoic acid**	Organic acids	HMDB0003681	18189	C02946
**Acetic acid**	Organic acids	HMDB0000042	176	C00033
**Azelaic acid**	Organic acids	HMDB0000784	2266	C08261
**Citramalic acid**	Organic acids	HMDB0000426	441696	C00815
**Formic acid**	Organic acids	HMDB0000142	284	C00058
**L-Lactic acid**	Organic acids	HMDB0000190	61503	C00186
**L-Malic acid**	Organic acids	HMDB0000156	222656	C00149
**N-Acetylputrescine**	Organic acids	HMDB0002064	122356	C02714
**Suberic acid**	Organic acids	HMDB0000893	10457	C08278

**Succinic acid**	Organic acids	HMDB0000254	1110	C00042
**Taurine**	Organic acids	HMDB0000251	1123	C00245
**2-Deoxy-D-Glucose**	Sugars		3865	C00586
**Alpha-D-Glucose**	Sugars	HMDB0003345	79025	C00267
**D-Galactosamine**	Sugars	METPA0263		C02262
**D-Mannose**	Sugars	HMDB0000169	18950	C00936
**Gluconic acid**	Sugars	HMDB0000625	10690	C00257
**Glyceric acid**	Sugars	HMDB0000139	439194	C00258
**L-Arabitol**	Sugars	HMDB0001851	94154	C00532
**Methyl beta-D-glucopyranoside**	Sugars	HMDB0029965	94214	
**myo-Inositol**	Sugars	HMDB0000211		C00137
**N-Acetylneuraminate**	Sugars		3568	C00270
**Ribitol**	Sugars	HMDB0000508		C00474

Feces				
**Metabolite**	**Chemical class**	**HMDB**	**PubChem**	**KEGG**
**Ciliatine**	Acides phosphoriques	HMDB0011747	339	C03557
**3-Dehydroshikimate**	Aliphatic compounds cyclic and acyclic	METPA0312		C02637
**Acetoin**	Aliphatic compounds cyclic and acyclic	HMDB0003243	4068	C00466
**Agmatine**	Aliphatic compounds cyclic and acyclic	HMDB0001432	199	C00179
**Cadaverine**	Aliphatic compounds cyclic and acyclic	HMDB0002322	273	C01672
**Quinic acid**	Aliphatic compounds cyclic and acyclic	HMDB0003072	6508	C00296
**Valerolactone**	Aliphatic compounds cyclic and acyclic	NA	10953	C02240
**Shikimic acid**	Aliphatic compounds cyclic and acyclic	HMDB0003070	8742	C00493
**Spermidine**	Aliphatic compounds cyclic and acyclic	HMDB0001257	1102	C00315
**3-Sulfinoalanine**	Amino acids-Peptides-Derivatives	HMDB0000996	439270	C00606
**L-Alanine**	Amino acids-Peptides-Derivatives	HMDB0000161	5950	C00041
**L-Cystathionine**	Amino acids-Peptides-Derivatives	HMDB0000099	439258	C02291
**N-Formylglycine**	Amino acids-Peptides-Derivatives		75606	
**Picolinic acid**	Amino acids-Peptides-Derivatives	HMDB0002243	1018	C10164
**Quinaldic acid**	Amino acids-Peptides-Derivatives	HMDB0000842	7124	C06325
**5-Hydroxy-L-tryptophan**	Amino acids-Peptides-Derivatives	HMDB0000472	144	C00643
**L-Dopa**	Amino acids-Peptides-Derivatives	HMDB0000181	6047	C00355
**DL-O-Phosphoserine**	Amino acids-Peptides-Derivatives	HMDB0001721	106	C01005

**Homocysteine**	Amino acids-Peptides-Derivatives	HMDB0000742	778	C00155
**S-Adenosylhomocysteine**	Amino acids-Peptides-Derivatives	HMDB0000939	439155	C00021
**2-Aminoisobutyric acid**	Amino acids-Peptides-Derivatives	HMDB0001906	6119	C03665
**3-Methoxytyrosine**	Amino acids-Peptides-Derivatives	HMDB0001434	1670	
**Diaminopimelic acid**	Amino acids-Peptides-Derivatives	HMDB0001370	99290	C00666
**3-Nitrotyrosine**	Amino acids-Peptides-Derivatives	HMDB0001904	65124	
**Cysteic acid**	Amino acids-Peptides-Derivatives	HMDB0002757	25701	C00506
**Hydroxykynurenine**	Amino acids-Peptides-Derivatives	HMDB0000732	89	C02794
**2-Hydroxyphenethylamine**	Aromatic compounds-Vitamins-Amines	HMDB0001065	1000	C02735
**3-Methoxytyramine**	Aromatic compounds-Vitamins-Amines	HMDB0000022	1669	C05587
**4-Hydroxyphenylpyruvic acid**	Aromatic compounds-Vitamins-Amines	HMDB0000707	979	C01179
**Dihydrouracil**	Aromatic compounds-Vitamins-Amines	HMDB0000076	649	C00429
**Isovaleric acid**	Aromatic compounds-Vitamins-Amines	HMDB0000718	10430	C08262
**L-Histidinol**	Aromatic compounds-Vitamins-Amines	HMDB0003431	165271	C00860
**p-Aminobenzoic acid**	Aromatic compounds-Vitamins-Amines	HMDB0001392	978	C00568
**Purine**	Aromatic compounds-Vitamins-Amines	HMDB0001366	1044	C15587
**Pyridoxine**	Aromatic compounds-Vitamins-Amines	HMDB0000239	1054	C00314
**Resorcinol monoacetate**	Aromatic compounds-Vitamins-Amines		5055	D02393
**Tyramine**	Aromatic compounds-Vitamins-Amines	HMDB0000306	5610	C00483
**Uracil-5-carboxylic acid**	Aromatic compounds-Vitamins-Amines		90301	C03030
**Vanillylmandelic acid**	Aromatic compounds-Vitamins-Amines	HMDB0000291	736172	C05584

**2,6-Dihydroxypyridine**	Aromatic compounds-Vitamins-Amines	METPA0354		C03056
**3-Hydroxybenzaldehyde**	Aromatic compounds-Vitamins-Amines		101	C03067
**5-Methylcytosine**	Aromatic compounds-Vitamins-Amines	HMDB0002894	65040	C02376
**Biliverdin**	Aromatic compounds-Vitamins-Amines	HMDB0001008	5353439	C00500
**Norepinephrine**	Aromatic compounds-Vitamins-Amines	HMDB0000216	439260	C00547
**4-Hydroxybenzoic acid**	Aromatic compounds-Vitamins-Amines	HMDB0000500	135	C00156
**Benzoic acid**	Aromatic compounds-Vitamins-Amines	HMDB0001870	243	C00539
**3-Amino-4-hydroxybenzoic acid**	Aromatic compounds-Vitamins-Amines		65083	C12115
**3-Amino-5-hydroxybenzoic acid**	Aromatic compounds-Vitamins-Amines		127115	C12107
**3-Hydroxyanthranilic acid**	Aromatic compounds-Vitamins-Amines	HMDB0001476	86	C00632
**3,4-Dihydroxymandelicacid**	Aromatic compounds-Vitamins-Amines		85782	C05580
**Ethyl-3-indole acetate**	Aromatic compounds-Vitamins-Amines			C00954
**Hydroquinone**	Aromatic compounds-Vitamins-Amines	HMDB0002434	785	C00530
**Vanylglycol**	Aromatic compounds-Vitamins-Amines	HMDB0001490	10805	C05594
**1H-Indole-3-acetamide**	Aromatic compounds-Vitamins-Amines	HMDB0029739	397	C02693
**Dimethylbenzimidazole**	Aromatic compounds-Vitamins-Amines	HMDB0003701	675	C03114
**Indoleacetaldehyde**	Aromatic compounds-Vitamins-Amines	HMDB0001190	800	C00637
**1-Naphthylamine**	Aromatic compounds-Vitamins-Amines	METPA1135		C14790
**5-Hydroxymethyluracil**	Aromatic compounds-Vitamins-Amines	HMDB0000469	78168	C03088
**Serotonin**	Aromatic compounds-Vitamins-Amines	HMDB0000259	5202	C00780
**Caprylic acid**	Lipids: Fatty acids-Steroids-Hormones-Vitamins	HMDB0000482	379	C06423

**Ergocalciferol**	Lipids: Fatty acids-Steroids-Hormones-Vitamins	HMDB0000900	5280793	C05441
**Lithocholic Acid**	Lipids: Fatty acids-Steroids-Hormones-Vitamins	HMDB0000761	11740284	C03990
**Maleic acid**	Lipids: Fatty acids-Steroids-Hormones-Vitamins	HMDB0000176	444972	C01384
**Mono-methylglutarate**	Lipids: Fatty acids-Steroids-Hormones-Vitamins		6993194	
**Oleic acid**	Lipids: Fatty acids-Steroids-Hormones-Vitamins	HMDB0000207	445639	C00712
**Rosmarinic acid**	Lipids: Fatty acids-Steroids-Hormones-Vitamins	HMDB0003572	5281792	C01850
**Sphinganine**	Lipids: Fatty acids-Steroids-Hormones-Vitamins	HMDB0000269	91486	C00836
**Vitamin A**	Lipids: Fatty acids-Steroids-Hormones-Vitamins	HMDB0000305	445354	C17276
**Petroselinic acid**	Lipids: Fatty acids-Steroids-Hormones-Vitamins	HMDB0002080	5461010	C08363
**All-trans-retinoic acid**	Lipids: Fatty acids-Steroids-Hormones-Vitamins	HMDB0001852	444795	C00777
**Chenodeoxycholic acid**	Lipids: Fatty acids-Steroids-Hormones-Vitamins	HMDB0000518	10133	C02528
**Cholesteryl acetate**	Lipids: Fatty acids-Steroids-Hormones-Vitamins	HMDB0003822	6427285	C02530
**2-Methylglutaric acid**	Lipids: Fatty acids-Steroids-Hormones-Vitamins	HMDB0000422	12046	
**7-Dehydrocholesterol**	Lipids: Fatty acids-Steroids-Hormones-Vitamins	HMDB0000032	439423	C01164
**Glycerol**	Lipids: Fatty acids-Steroids-Hormones-Vitamins	HMDB0000131	753	C00116
**Alpha-Tocopherol**	Lipids: Fatty acids-Steroids-Hormones-Vitamins	HMDB0001893	14985	C02477
**Elaidic acid**	Lipids: Fatty acids-Steroids-Hormones-Vitamins	HMDB0000573	637517	C01712
**Cortexolone**	Lipids: Fatty acids-Steroids-Hormones-Vitamins	HMDB0000015	53477676	C05488
**Deoxycorticosterone acetate**	Lipids: Fatty acids-Steroids-Hormones-Vitamins		5952	C14554
**Valeric acid**	Lipids: Fatty acids-Steroids-Hormones-Vitamins	HMDB0000892	7991	C00803
**5'-Deoxyadenosine**	Nucleotides-Nucleosides-Derivatives	HMDB0001983	439182	C05198

**dCMP**	Nucleotides-Nucleosides-Derivatives	HMDB0001202	13945	C00239
**Cytidine2',3'-cyclicphosphate**	Nucleotides-Nucleosides-Derivatives		5401	C02354
**5'UMP**	Nucleotides-Nucleosides-Derivatives		3405	C00105
**Cytidine**	Nucleotides-Nucleosides-Derivatives	HMDB0000089	6253	C00475
**Cyclic GMP**	Nucleotides-Nucleosides-Derivatives	HMDB0001314	24316	C00942
**Deoxyuridine**	Nucleotides-Nucleosides-Derivatives	HMDB0000012	13712	C00526
**Dimethylsulfone**	Organic acids	HMDB0004983	6213	C11142
**Malonic acid**	Organic acids	HMDB0000691	867	C04025
**Pimelic acid**	Organic acids	HMDB0000857	385	C02656
**4-Heptenal diethyl acetal**	Organic acids	HMDB0032306	97750	C04067
**Isobutyric acid**	Organic acids	HMDB0001873	6590	C02632
**D-Maltose**	Sugars	HMDB0000163	10991489	C00208
**Rhamnose**	Sugars	HMDB0000849	25310	C00507
**D-Lyxose**	Sugars		3759	C00476
**2-Acetamido-2-deoxy-beta-glucosylamine**	Sugars			
**Glucosamine**	Sugars	HMDB0001514	439213	C00329
**D-Ribose**	Sugars	HMDB0000283	5779	C00121
**2-Amino-2-deoxy-D-gluconate**	Sugars	METPA0433		C03752

Feces Plasma				
**Metabolite**	Chemical Class	HMDB	PubChem	KEGG
**Diethanolamine**	Aliphatic compounds cyclic and acyclic	HMDB0004437	8113	C06772
**Putrescine**	Aliphatic compounds cyclic and acyclic	HMDB0001414	1045	C00134
**4-Guanidinobutanoic acid**	Amino acids-Peptides-Derivatives	HMDB0003464	500	C01035
**5-Aminopentanoic acid**	Amino acids-Peptides-Derivatives	HMDB0003355	138	C00431
**Aminoadipic acid**	Amino acids-Peptides-Derivatives	HMDB0000510	469	C00956
**Carnosine**	Amino acids-Peptides-Derivatives	HMDB0000033	439224	C00386
**L-Norvaline**	Amino acids-Peptides-Derivatives		4949	C01826
**N-Acetyl-L-aspartic acid**	Amino acids-Peptides-Derivatives	HMDB0000812	65065	C01042
**N-Acetylglutamic acid**	Amino acids-Peptides-Derivatives	HMDB0001138	185	C00624
**N-Alpha-acetyllysine**	Amino acids-Peptides-Derivatives	HMDB0000446	192590	C12989
**2',4'-Dihydroxyacetophenone**	Aromatic compounds-Vitamins-Amines	HMDB0029659	6990	C03663
**2-Aminobenzoic acid**	Aromatic compounds-Vitamins-Amines	HMDB0001123	227	C00108
**3-Hydroxybenzoic acid**	Aromatic compounds-Vitamins-Amines	HMDB0002466	7420	C00587
**Caffeic acid**	Aromatic compounds-Vitamins-Amines	HMDB0001964	1549111	C01481
**Histamine**	Aromatic compounds-Vitamins-Amines	HMDB0000870	774	C00388
**Hydroxyphenyllactic acid**	Aromatic compounds-Vitamins-Amines	HMDB0000755	9378	C03672
**Indole-3-methyl acetate**	Aromatic compounds-Vitamins-Amines	HMDB0029738	74706	

**Phenylacetaldehyde**	Aromatic compounds-Vitamins-Amines	HMDB0006236	998	C00601
**Pyridoxamine**	Aromatic compounds-Vitamins-Amines	HMDB0001431	1052	C00534
**Salicylamide**	Aromatic compounds-Vitamins-Amines	HMDB0015687	5147	NA
**Tryptamine**	Aromatic compounds-Vitamins-Amines	HMDB0000303	1150	C00398
**17a-Estradiol**	Lipids: Fatty acids-Steroids-Hormones-Vitamins	HMDB0000429	68570	C02537
**2,3-Dihydroxypropyl tetradecanoate**	Lipids: Fatty acids-Steroids-Hormones-Vitamins		79050	
**21-Tetrahydroxy-5-alpha-pregnan-20-one**	Lipids: Fatty acids-Steroids-Hormones-Vitamins		5864	
**3-Methylglutaric acid**	Lipids: Fatty acids-Steroids-Hormones-Vitamins	HMDB0000752	12284	
**Butyric acid**	Lipids: Fatty acids-Steroids-Hormones-Vitamins	HMDB0000039	264	C00246
**Cortisone**	Lipids: Fatty acids-Steroids-Hormones-Vitamins	HMDB0002802	225609	C00762
**Deoxycholic acid**	Lipids: Fatty acids-Steroids-Hormones-Vitamins	HMDB0000626	222528	C04483
**Dihydroxy-beta-cholanate**	Lipids: Fatty acids-Steroids-Hormones-Vitamins			
**Gamma-Linolenic acid**	Lipids: Fatty acids-Steroids-Hormones-Vitamins	HMDB0003073	5280933	C06426
**Glyceryl monoacetate**	Lipids: Fatty acids-Steroids-Hormones-Vitamins		33510	
**Leukotriene B4**	Lipids: Fatty acids-Steroids-Hormones-Vitamins	HMDB0001085	5283128	C02165
**Deoxyadenosine**	Nucleotides-Nucleosides-Derivatives	HMDB0000101	13730	C00559
**Adipic acid**	Organic acids	HMDB0000448	196	C06104
**Isocitric acid**	Organic acids	HMDB0000193	1198	C00311
**Propionic acid**	Organic acids	HMDB0000237	1032	C00163
**D-Glucuronic acid**	Sugars	HMDB0000127	94715	C00191

List of metabolites according to the platform

Table S5: Metabolites detected by platform in plasma, milk and feces. In bold the platform where the metabolite was annotated with the lowest CV (best reproducibility)

**Plasma** (Blood :MeOH : $CHCl_3$ 1 :1 :1)	
**Metabolite**	**Analytical platform**
3-Hydroxycapric acid	RP+
1-hydroxy-2-naphthoate	HILIC-
1-Methyladenosine	RP- **HILIC+**
Glyceryl monoacetate	HILIC+
2',4'-Dihydroxyacetophenone	RP+
2,4-Dihydroxypyrimidine-5-carboxylic acid	HILIC-
Gentisic acid	HILIC-
2-Aminophenol	HILIC+
Deoxyadenosine	NMR
2-Deoxy-D-Glucose	RP+
Deoxyguanosine	HILIC-
2-Hydroxybutyric acid	RP- **NMR**
Ortho-Hydroxyphenylacetic acid	HILIC-
Ketoleucine	RP- **NMR**

Protocatechuic acid	HILIC-
3,4-Dihydroxybenzeneacetic acid	HILIC-
3-(2-Hydroxyphenyl)propanoic acid	**RP-** HILIC-
Hydroxyphenyllactic acid	HILIC-
21-Tetrahydroxy-5-alpha-pregnan-20-one	HILIC-
Dihydroxy-beta-cholanate	HILIC-
3-Hydroxymethylglutaric acid	HILIC-
3-Hydroxybenzoic acid	HILIC-
3-Hydroxybutyric acid	NMR
(S)-3-Hydroxyisobutyric acid	HILIC- **NMR**
3-Hydroxyphenylacetic acid	HILIC-
3-Methylglutaric acid	RP-
3-Methyldioxyindole	RP-
Ureidopropionic acid	RP-
4-Acetamidobutanoic acid	RP-
Gamma-Aminobutyric acid	RP+
4-Hydroxycinnamic acid	HILIC+
4-Guanidinobutanoic acid	RP+
4-Hydroxy-L-proline	RP+
p-Hydroxyphenylacetic acid	HILIC-
5-Aminolevulinic acid	RP+
5-Aminopentanoic acid	RP+

5-Hydroxyindoleacetic acid	**RP+ HILIC-**
5'-Methylthioadenosine	RP+ **HILIC+**
Pyroglutamic acid	RP+
Acetic acid	NMR
Acetoacetic acid	HILIC-
Acetone	NMR
N-Acetyl-L-alanine	RP+
N-Acetyl-L-aspartic acid	HILIC+
N-Acetylglutamic acid	RP+
Acetylglycine	RP-
N-Acetylleucine	RP+
N-Acetylneuraminate	RP-
N-Acetyl-L-phenylalanine	**RP-** HILIC-
N-Acetylputrescine	HILIC+
N-Acetylserine	RP-
N-Acetylserotonin	HILIC+
N-acetyltryptophan	RP-
Adenine	RP+
Adenosine	HILIC-
Adipic acid	RP+
Allantoin	**RP-** HILIC+ NMR
Allose	HILIC-

Alpha-D-Glucose	RP- HILIC- **NMR**
Aminoadipic acid	RP-
2-Aminobenzoic acid	HILIC+
L-Arabitol	HILIC-
L-Arginine	RP-
L-Asparagine	**RP-** HILIC+
L-Aspartic acid	RP-
Azelaic acid	RP-
Benzyl alcohol	RP- **HILIC-**
Beta-Alanine	RP+ **NMR**
Betaine	RP+
Bilirubin	HILIC+
Biotin	RP+
Butyric acid	NMR
Caffeic acid	HILIC-
L-Carnitine	RP+
Carnosine	RP- **NMR**
Pyrocatechol	RP-
Choline	NMR
trans-Cinnamic acid	RP+ **HILIC-**
Citramalic acid	RP-
Citric acid	NMR

Citrulline	RP-
Cortisone	HILIC-
Creatine	**RP- NMR**
Creatinine	NMR
Cyclohexane-1,2-Diol	HILIC+
L-Cystine	RP-
Cytosine	RP+
4-Trimethylammoniobutanoic acid	RP+
Deoxycholic acid	HILIC-
Deoxycytidine	HILIC-
Diethanolamine	HILIC+
Dihydroxyacetone phosphate	HILIC-
N,N-Dimethylphenethylamine	HILIC+
Dimethylamine	NMR
Dimethylglycine	NMR
17a-Estradiol	HILIC+
O-Phosphoethanolamine	RP-
Ethylmalonic acid	RP-
Formic acid	NMR
Formyl-L-methionylpeptide	RP-
Galactitol	HILIC-
D-Galactosamine	RP+

D-Galactose	HILIC-
D-Glucuronic acid	RP-
L-Glutamic acid	RP-
L-Glutamine	RP+
Glyceric acid	RP-
Glycerophosphocholine	RP+
Glycine	RP+ **NMR**
Glycocholic acid	RP-
Glycolaldehyde dimer	HILIC+
Guanidoacetic acid	RP+ **HILIC+**
Guanine	HILIC-
Guanosine	HILIC+
Guanosine monophosphate	RP+
Caproic acid	HILIC+
Hippuric acid	RP- **HILIC-** NMR
Histamine	HILIC+
L-Histidine	RP- **NMR**
Homogentisic acid	HILIC-
L-Homoserine	RP-
Homovanillic acid	HILIC-
Hypoxanthine	RP-
Indoleacetic acid	**RP+** HILIC-

Indoxyl sulfate	**RP-** HILIC-
Inosine	RP+ **HILIC+**
Isocitric acid	RP-
L-Isoleucine	NMR
N6-(delta2-Isopentenyl)-adenine	RP-
L-Kynurenine	RP+
L-Lactic acid	RP- **NMR**
Alpha-Lactose	NMR
L-Leucine	NMR
L-Norleucine	RP+
Leukotriene B4	RP+
Gamma-Linolenic acid	HILIC+
Lumichrome	**RP+** HILIC+
L-Lysine	RP+ **NMR**
L-Malic acid	RP-
D-Mannose	**HILIC+** NMR
L-Methionine	**RP-** NMR
Methyl beta-D-glucopyranoside	HILIC-
Indole-3-methyl acetate	RP+ **HILIC+**
Methyl jasmonate	HILIC+
Methylguanidine	HILIC+
3-Methylhistidine	NMR

Methylmalonic acid	HILIC-
myo-Inositol	HILIC-
N-Alpha-acetyllysine	RP+
L-Norvaline	RP+
Acetylcholine	NMR
12-Hydroxydodecanoic acid	RP- **HILIC-**
Ornithine	RP+
Orotic acid	HILIC-
Pantothenic acid	HILIC+
Paraxanthine	RP+ **HILIC+**
Phenylacetaldehyde	HILIC-
Phenylacetic acid	HILIC-
L-Phenylalanine	RP+ **HILIC+**
Phenylpyruvic acid	HILIC+
Phosphorylcholine	NMR
Pipecolic acid	RP- **HILIC+**
L-Proline	RP+
Propanal	HILIC+
Propionic acid	NMR
Putrescine	RP+
Pyridoxamine	RP+
Pyruvic acid	NMR

2,3-Dihydroxypropyl tetradecanoate	RP+ **HILIC+**
Ribitol	HILIC-
Salicylamide	HILIC+
Sarcosine	NMR
L-Serine	RP+
Sorbose	HILIC-
Suberic acid	RP-
Succinic acid	RP-
Taurine	RP-
Lithocholyltaurine	HILIC-
Theobromine	HILIC+
L-Threonine	RP+ **NMR**
Thymidine	**HILIC+** HILIC-
Thyroxine	RP-
Trigonelline	HILIC+
Trimethylamine	HILIC+
Tryptamine	RP+
L-Tryptophan	**RP-** HILIC+ NMR
L-Tyrosine	RP- **HILIC+** NMR
Uracil	**RP+** HILIC+
Uric acid	**RP+** HILIC-
Uridine	**RP-** HILIC+

Urocanic acid	RP+
L-Valine	RP+ **HILIC+** NMR
Xanthine	**RP+** HILIC-
Xanthosine	HILIC-

**Milk**<br>(Milk :H_2O :MeOH :$CHCl_3$ 1 :3 :4 :6)	
**Metabolites**	**Analytical platform**
3-Hydroxycapric acid	**RP-** HILIC+
1-Aminocyclopropanecarboxylic acid	RP+
1-Methyladenosine	HILIC+
2,4-Dihydroxypyrimidine-5-carboxylic acid	HILIC-
Gentisic acid	HILIC-
2-Aminophenol	HILIC+
2-Deoxy-D-Glucose	**RP+ HILIC-**
Deoxyguanosine	HILIC-
2-Hydroxybutyric acid	RP-
Ortho-Hydroxyphenylacetic acid	HILIC-
2-Hydroxypyridine	HILIC+
Citraconic acid	HILIC-
Oxoglutaric acid	NMR
Protocatechuic acid	HILIC-

3,4-Dihydroxybenzeneacetic acid	HILIC-
Cyclic AMP	HILIC+
3-(2-Hydroxyphenyl)propanoic acid	**RP-** HILIC-
3-Aminoisobutanoic acid	HILIC-
3-Hydroxymethylglutaric acid	HILIC-
3-Hydroxybutyric acid	NMR
3-Hydroxyphenylacetic acid	HILIC-
3-Methyldioxyindole	RP+
3-Methyladenine	RP+ **HILIC-**
Ureidopropionic acid	RP-
4-Acetamidobutanoic acid	**RP-** HILIC+
Gamma-Aminobutyric acid	**RP+** HILIC+
Kynurenic acid	RP+
4-Hydroxybenzaldehyde	HILIC-
Oxfenicine	RP+
4-Hydroxy-L-proline	**RP+** HILIC+
p-Hydroxyphenylacetic acid	**RP-** HILIC-
Imidazoleacetic acid	HILIC-
Ketoleucine	**RP-** HILIC-
4-Methylcatechol	RP-
5-Aminolevulinic acid	**RP+** HILIC+
5-Hydroxyindoleacetic acid	HILIC+

5-Hydroxylysine	RP+
5'-Methylthioadenosine	**RP+** HILIC+
Pyroglutamic acid	RP-
L-Fucose	HILIC-
Acetamide	NMR
Acetic acid	NMR
Acetoacetic acid	**HILIC-** NMR
N-Acetyl-L-alanine	RP+
L-Acetylcarnitine	**HILIC+** NMR
N-Acetylgalactosamine	HILIC-
N-Acetyl-D-glucosamine	HILIC+
Acetylglycine	RP+
N-Acetylleucine	HILIC-
N-Acetylmannosamine	RP+ **HILIC-**
N-Acetyl-L-methionine	RP-
N-Acetylneuraminate	RP-
N-Acetyl-L-phenylalanine	**RP-** HILIC-
N-Acetylputrescine	HILIC+
N-Acetylserine	RP-
N-Acetylserotonin	HILIC+
N-acetyltryptophan	RP-
Adenine	HILIC+

Adenosine	**RP+** HILIC-
ADP-glucose	RP-
Adenosine monophosphate	RP-
Allantoin	RP- **HILIC+**
Allose	HILIC-
L-Allothreonine	HILIC+
Galactose 1-phosphate	HILIC+
Alpha-D-Glucose	**RP-** HILIC- NMR
Anserine	RP+
L-Arabitol	RP- **HILIC-**
L-Arginine	**RP+** HILIC-
L-Asparagine	**RP+** HILIC-
L-Aspartic acid	RP-
Azelaic acid	RP-
Benzyl alcohol	HILIC-
Beta-Alanine	**RP+** HILIC+ NMR
Betaine	**RP+** HILIC+
Biotin	**RP-** HILIC+
Butanal	HILIC+
L-Carnitine	**RP+** HILIC+
Pyrocatechol	RP-
Cellobiose	HILIC-

Cholic acid	HILIC-
Choline	RP+ **NMR**
trans-Cinnamic acid	RP+
Citramalic acid	HILIC+
Citric acid	NMR
Citrulline	**RP-** HILIC-
Corticosterone	RP+
Cortisol	RP+
Cortisol-21-acetate	RP+
Creatine	RP+ HILIC+ **NMR**
Phosphocreatine	NMR
Creatinine	**RP-** HILIC+
Cyclohexane-1,2-Diol	HILIC+
Cytosine	RP+ **HILIC+**
4-Trimethylammoniobutanoic acid	**RP+** HILIC+
Deoxycytidine	HILIC+
Dethiobiotin	RP+
Diacetyl	HILIC+
L-Dihydroorotic acid	RP+
Dimethyl sulfone	NMR
N,N-Dimethylphenethylamine	HILIC+
Dimethylamine	NMR

Epinephrine	RP+
Ethanolamine	HILIC+
O-Phosphoethanolamine	RP-
Ethylmalonic acid	HILIC-
trans-Ferulic acid	**RP-** HILIC+
Formic acid	NMR
Formyl-L-methionylpeptide	RP-
D-Fructose	HILIC-
Fumaric acid	NMR
Galactaric acid	RP-
Galactitol	HILIC-
D-Galactosamine	RP+
D-Galactose	HILIC-
Gluconic acid	RP-
Glucosamine 6-phosphate	RP-
Glucose 6-phosphate	RP-
L-Glutamic acid	**RP+** HILIC-
L-Glutamine	**RP+** HILIC+
Glyceraldehyde-3-phosphatediethylacetal	RP+
Glyceric acid	RP-
Glycerol 3-phosphate	HILIC+
Glycerophosphocholine	**RP+** HILIC+

Glycine	**RP+** HILIC+
Glycocholic acid	RP-
Glycolaldehyde dimer	HILIC+
Guanidoacetic acid	RP+ **HILIC+**
Guanine	HILIC+
Guanosine	**RP+** HILIC+
Heptanoic acid	HILIC+
Caproic acid	HILIC+
Hippuric acid	**RP-** HILIC+ NMR
L-Histidine	RP-
Homogentisic acid	HILIC+
L-Homoserine	**RP-** HILIC-
Homovanillic acid	HILIC-
Hypotaurine	HILIC-
Hypoxanthine	RP- **HILIC+**
Indoleacetic acid	RP+
Tryptophanol	RP+ **HILIC-**
Indolepyruvate	HILIC+
Indoxyl sulfate	**RP-** HILIC-
Inosine	**RP-** HILIC+ NMR
L-Isoleucine	HILIC+
N6-(delta2-Isopentenyl)-adenine	RP-

L-Kynurenine	RP+
L-Lactic acid	RP- HILIC- **NMR**
Alpha-Lactose	NMR
L-Leucine	HILIC+
Lumichrome	**RP+** HILIC-
L-Lysine	RP+
L-Malic acid	RP-
Mannitol	**RP-** HILIC-
D-Mannose	HILIC+
Melatonin	HILIC+
L-Methionine	RP-
Methyl beta-D-glucopyranoside	HILIC-
Methylguanidine	HILIC+
3-Methylhistidine	RP+
Methylmalonic acid	RP+
N-Methyltryptamine	**RP+** HILIC+
myo-Inositol	RP+ HILIC- **NMR**
N-Trimethyllysine	**RP+** HILIC+
Niacinamide	**RP+** HILIC+
Nicotinic acid	RP-
L(-)-Nicotine	HILIC+
N-Methylhydantoin	NMR

L-Norleucine	HILIC+
Phenylacetylglycine	NMR
Acetylcholine	NMR
12-Hydroxydodecanoic acid	RP-
Ornithine	RP+ **HILIC-**
Orotic acid	HILIC- **NMR**
Palatinose	HILIC-
Pantolactone	HILIC-
Pantothenic acid	**RP-** HILIC+
Paraxanthine	HILIC+
L-Phenylalanine	**RP-** HILIC+
Phosphorylcholine	RP+ **NMR**
Pipecolic acid	HILIC+
L-Proline	RP+ **HILIC+**
Propanal	HILIC+
Pterin	RP+
Pyrrole-2-carboxylic acid	HILIC+
Ribitol	HILIC-
Riboflavin	HILIC+
Glucaric acid	RP-
Salicyluric acid	HILIC+
Sarcosine	HILIC+

L-Serine	**RP-** HILIC+
Sorbitol	HILIC-
L-Sorbose	HILIC-
Suberic acid	RP-
Succinic acid	RP- HILIC+ **NMR**
Sucrose	HILIC-
D-Tagatose	HILIC-
Taurine	RP- **HILIC+** NMR
Lithocholyltaurine	HILIC-
Theobromine	HILIC+
Theophylline	HILIC+
L-Threonine	**RP+** HILIC-
Thymidine	HILIC-
Thymine	**RP+** HILIC+
trans-Aconitic acid	RP- **HILIC-** NMR
Trigonelline	**RP+** HILIC+
Trimethylamine	HILIC+
L-Tryptophan	**RP-** HILIC+
L-Tyrosine	**RP+** HILIC+
Uracil	**RP-** HILIC+
Uric acid	**RP+** HILIC-
Uridine	**RP-** NMR

Uridine diphosphategalactose	HILIC- **NMR**
Uridine diphosphate glucose	HILIC- **NMR**
Uridine diphosphate-N-acetylgalactosamine	HILIC-
Uridine-5-diphospho-n-acetylglucosamine	HILIC- **NMR**
Urocanic acid	**RP+** HILIC-
L-Valine	RP+
Xanthine	**RP-** HILIC-
D-Xylitol	HILIC-

**FECES** (MeOH : H_2O 4 :1)	
**Metabolites**	**Analytical platform**
3-Hydroxycapric acid	RP+
1-Aminocyclopropanecarboxylic acid	**RP+** HILIC +
1-Methyladenosine	**RP+** HILIC +
1-Naphthylamine	HILIC+
Glyceryl monoacetate	HILIC+
2',4'-Dihydroxyacetophenone	HILIC-
2,4-Dihydroxypyrimidine-5-carboxylic acid	HILIC-
Gentisic acid	**RP- HILIC-**
Diaminopimelic acid	RP+

2,6-Dihydroxypyridine	HILIC+
2-Acetamido-2-deoxy-beta-glucosylamine	**RP+** HILIC-
2-Aminoisobutyric acid	HILIC+
Ciliatine	RP-
2-Aminophenol	**RP+** HILIC-
Deoxyadenosine	RP- **HILIC+**
dCMP	RP-
2-Deoxy-D-Glucose	**RP+** HILIC-
Deoxyguanosine	HILIC-
2-Hydroxybutyric acid	RP-
Ortho-Hydroxyphenylacetic acid	HILIC- NMR
2-Hydroxypyridine	HILIC+
2-Methylglutaric acid	HILIC-
Citraconic acid	HILIC-
Quinaldic acid	HILIC-
Protocatechuic acid	HILIC-
3,4-Dihydroxybenzeneacetic acid	HILIC-
L-Dopa	**RP+** HILIC+
3-(2-Hydroxyphenyl)propanoic acid	**RP-** HILIC-
Hydroxyphenyllactic acid	RP-
4-Hydroxyphenylpyruvic acid	RP+

21-Tetrahydroxy-5-alpha-pregnan-20-one	**RP+** HILIC-
Dihydroxy-beta-cholanate	HILIC-
Lithocholic Acid	RP-
3-Amino-4-hydroxybenzoic acid	HILIC+
3-Amino-5-hydroxybenzoic acid	RP+
3-Aminoisobutanoic acid	HILIC-
3-Dehydroshikimate	RP-
3-Hydroxyanthranilic acid	**RP+** HILIC+
3-Hydroxybenzaldehyde	HILIC-
3-Hydroxybenzoic acid	RP- **HILIC-**
Hydroxykynurenine	RP+
3-Hydroxyphenylacetic acid	**RP-** HILIC-
Vanillylmandelic acid	**RP-** HILIC+
3-Methoxytyrosine	HILIC+
3-Methoxytyramine	RP+ **HILIC-**
3-Methylglutaric acid	**RP- HILIC-**
3-Methyldioxyindole	RP- **HILIC+**
3-Methyladenine	RP+ **HILIC+**
3-Nitrotyrosine	RP-
3-Sulfinoalanine	RP-
Ureidopropionic acid	**RP+** HILIC+

4-Acetamidobutanoic acid	HILIC-
p-Aminobenzoic acid	HILIC-
4-Guanidinobutanoic acid	**RP+** HILIC+
Kynurenic acid	**RP+** HILIC+
Vanylglycol	HILIC+
4-Hydroxybenzaldehyde	HILIC-
4-Hydroxybenzoic acid	HILIC-
Oxfenicine	RP+
4-Hydroxy-L-proline	**RP+** HILIC+
p-Hydroxyphenylacetic acid	**RP+** HILIC-
Imidazoleacetic acid	RP+ **HILIC+**
Ketoleucine	RP- **HILIC-**
Dihydrouracil	**RP+** HILIC+
5-Aminolevulinic acid	RP+
5-Aminopentanoic acid	RP+ **HILIC+**
5'-Deoxyadenosine	HILIC+
5-Hydroxyindoleacetic acid	**RP+** HILIC-
5-Hydroxylysine	RP+
5-Hydroxymethyluracil	RP-
5-Hydroxy-L-tryptophan	RP-
5-Methylcytosine	**RP+ HILIC+**

5'-Methylthioadenosine	RP+ **HILIC+**
Pyroglutamic acid	RP- **HILIC-**
Valerolactone	HILIC+
Pimelic acid	HILIC-
Acetamide	NMR
Acetic acid	NMR
Acetoin	HILIC-
N-Acetyl-L-alanine	RP+
N-Acetyl-L-aspartic acid	RP-
L-Acetylcarnitine	**HILIC+** NMR
N-Acetylgalactosamine	HILIC-
N-Acetyl-D-glucosamine	**RP-** HILIC+
N-Acetylglutamic acid	RP+
Acetylglycine	RP-
N-Acetylleucine	RP+
N-Acetylmannosamine	**RP+ HILIC+**
N-Acetyl-L-methionine	RP- **HILIC+**
N-Acetylneuraminate	RP+
N-Acetyl-L-phenylalanine	RP+ **HILIC+**
N-Acetylputrescine	RP+ **HILIC+**
N-Acetylserine	RP+

N-Acetylserotonin	**RP-** HILIC+
N-acetyltryptophan	RP+
Adenine	**RP+** HILIC+
Adenosine	RP+ **HILIC+**
Cyclic AMP	HILIC-
S-Adenosylhomocysteine	HILIC+
Adipic acid	**RP-** HILIC-
Agmatine	RP+
L-Alanine	HILIC+
Allantoin	RP-
L-Allothreonine	HILIC+
Alpha-D-Glucose	HILIC-
Alpha-Tocopherol	HILIC+
Aminoadipic acid	**RP+** HILIC+
Anserine	HILIC+
2-Aminobenzoic acid	HILIC-
L-Arabitol	RP-
L-Arginine	**RP+ HILIC+**
L-Asparagine	**RP+** HILIC-
L-Aspartic acid	RP+
Azelaic acid	**RP-** HILIC-

Benzoic acid	HILIC-
Benzyl alcohol	HILIC-
Beta-Alanine	**RP+** HILIC+ NMR
Betaine	**RP+** HILIC+ NMR
Biliverdin	HILIC+
Biotin	RP+ **HILIC+**
Butyric acid	HILIC- **NMR**
Cadaverine	RP+ **HILIC+**
Caffeic acid	RP-
Caprylic acid	HILIC-
L-Carnitine	**RP+** HILIC+
Carnosine	RP+ **HILIC+**
Pyrocatechol	**RP-** HILIC-
Chenodeoxycholic acid	HILIC-
Cholic acid	RP+
7-Dehydrocholesterol	HILIC+
Cholesteryl acetate	HILIC+
Choline	RP+ **NMR**
trans-Cinnamic acid	**RP+** HILIC-
Citramalic acid	**RP-** HILIC+
Citrulline	**RP+** HILIC+

Corticosterone	HILIC+
Cortisol	RP+
Cortisol-21-acetate	RP-
Cortisone	HILIC-
Creatine	RP+
Creatinine	**RP+** HILIC+
Cyclohexane-1,2-Diol	HILIC-
L-Cystathionine	RP-
Cysteic acid	**RP-** HILIC-
Cytidine	**RP+** HILIC+
Cytidine2',3'-cyclicphosphate	RP-
Cytosine	RP+ **HILIC+**
4-Trimethylammoniobutanoic acid	**RP+** HILIC+
Deoxycholic acid	HILIC-
Deoxycorticosterone acetate	RP+
Deoxycytidine	RP- **HILIC+**
Deoxyuridine	HILIC+
Diacetyl	HILIC+
Diethanolamine	**RP+** HILIC+
4-Heptenal diethyl acetal	HILIC-
L-Dihydroorotic acid	RP-

3,4-Dihydroxymandelicacid	RP-
N,N-Dimethylphenethylamine	HILIC - **NMR**
Dimethylbenzimidazole	RP+
Elaidic acid	HILIC-
Epinephrine	RP+
17a-Estradiol	HILIC+
Ethanolamine	HILIC+
O-Phosphoethanolamine	RP+
Ethyl-3-indole acetate	HILIC-
Ethylmalonic acid	**RP-** HILIC-
trans-Ferulic acid	HILIC-
Formic acid	NMR
N-Formylglycine	RP+
Formyl-L-methionylpeptide	RP+
Fumaric acid	RP-
Galactitol	RP+
D-Galactosamine	RP+
Gluconic acid	RP-
2-Amino-2-deoxy-D-gluconate	RP-
Glucosamine	RP- **HILIC+**
Glucosamine 6-phosphate	RP-

Glucose 6-phosphate	RP-
D-Glucuronic acid	RP-
L-Glutamic acid	**RP+** HILIC+
L-Glutamine	**RP+** HILIC-
Glyceraldehyde-3-phosphatediethylacetal	RP-
Glyceric acid	RP-
Glycerol	NMR
Glycerophosphocholine	RP+ **HILIC+**
Glycine	**RP+** HILIC+ NMR
Glycocholic acid	RP-
Guanidoacetic acid	HILIC+
Guanine	RP- **HILIC+**
Guanosine	RP+ **HILIC+**
Cyclic GMP	RP-
Caproic acid	HILIC-
Hippuric acid	RP- **HILIC+**
Histamine	RP+ **HILIC+**
L-Histidine	**RP+** HILIC+
L-Histidinol	HILIC+
Homocysteine	RP+
Homogentisic acid	RP+

L-Homoserine	RP-
Homovanillic acid	HILIC-
Hydroquinone	HILIC-
Hypoxanthine	**RP+** HILIC+
Indoleacetaldehyde	HILIC+
1H-Indole-3-acetamide	RP+ **HILIC+**
Indoleacetic acid	RP+ **HILIC-**
Tryptophanol	HILIC+
Indolepyruvate	HILIC-
Indoxyl sulfate	HILIC-
Inosine	**RP-** HILIC-
Isobutyric acid	HILIC- **NMR**
Isocitric acid	RP-
L-Isoleucine	HILIC+ **NMR**
N6-(delta2-Isopentenyl)-adenine	RP- **HILIC+**
Isovaleric acid	NMR
L-Kynurenine	RP-
L-Lactic acid	RP- HILIC- **NMR**
L-Leucine	HILIC+
L-Norleucine	RP+
Leukotriene B4	**RP+** HILIC-

Gamma-Linolenic acid	HILIC+
Lumichrome	**RP+** HILIC+
L-Lysine	**RP+** HILIC+
D-Lyxose	**RP+** HILIC-
L-Malic acid	RP-
Maleic acid	HILIC-
Malonic acid	RP-
D-Maltose	RP-
Mannitol	RP-
D-Mannose	HILIC+
L-Methionine	**RP+** HILIC+
Methyl beta-D-glucopyranoside	HILIC-
Methylguanidine	HILIC+
3-Methylhistidine	RP+
Indole-3-methyl acetate	**RP+** HILIC+
N-Methyltryptamine	**RP+** HILIC+
Mono-methylglutarate	HILIC-
myo-Inositol	HILIC-
N-Alpha-acetyllysine	**RP-** HILIC+
N-Trimethyllysine	**RP+** HILIC+
Niacinamide	**RP+** HILIC+

Nicotinic acid	**RP+** HILIC+
Norepinephrine	RP+
L-Norvaline	RP+ **HILIC+**
Acetylcholine	NMR
Oleic acid	RP+ **HILIC+**
12-Hydroxydodecanoic acid	**RP+** HILIC-
Ornithine	RP+ **HILIC+**
Orotic acid	RP+ **HILIC-**
Pantolactone	HILIC-
Pantothenic acid	**RP+ HILIC+**
Paraxanthine	HILIC+
Valeric acid	NMR
Petroselinic acid	HILIC-
Phenylacetaldehyde	HILIC-
L-Phenylalanine	**RP+** HILIC+ NMR
2-Hydroxyphenethylamine	HILIC+
Phosphorylcholine	NMR
DL-O-Phosphoserine	RP-
Picolinic acid	HILIC+
Pipecolic acid	RP+
L-Proline	**RP+** HILIC+

Propanal	HILIC+
Propionic acid	NMR
Pterin	**RP+** HILIC+
Purine	RP+ **HILIC+**
Putrescine	RP+
Pyridoxamine	RP+
Pyridoxine	RP+
Pyrrole-2-carboxylic acid	**RP+** HILIC-
Quinic acid	RP-
2,3-Dihydroxypropyl tetradecanoate	RP+
Cortexolone	HILIC+
Resorcinol monoacetate	HILIC-
All-trans-retinoic acid	RP+ **HILIC+**
Vitamin A	HILIC+
Rhamnose	HILIC-
Ribitol	HILIC-
Riboflavin	RP+ **HILIC+**
D-Ribose	HILIC-
Rosmarinic acid	HILIC-
Salicylamide	**RP- HILIC-**
Salicyluric acid	HILIC+

Sarcosine	HILIC-
L-Serine	**RP- HILIC+**
Serotonin	RP+ **HILIC+**
Shikimic acid	HILIC+
Spermidine	RP+ **HILIC+**
Sphinganine	RP+
Suberic acid	**RP-** HILIC-
Succinic acid	**RP-** HILIC+ NMR
Taurine	**RP- HILIC-**
L-Threonine	**RP+** HILIC+ NMR
Thymidine	RP- **HILIC-**
Thymine	RP+ **HILIC-**
trans-Aconitic acid	RP-
Trigonelline	**RP+** HILIC+
Trimethylamine	HILIC+
Tryptamine	HILIC+
L-Tryptophan	**RP+** HILIC+
Tyramine	HILIC+
L-Tyrosine	**RP+** HILIC- NMR
Uracil	RP+ **HILIC-**
Uracil-5-carboxylic acid	RP+

Uric acid	RP+
Uridine	**RP+** HILIC+
5'UMP	HILIC+
Urocanic acid	RP+ **HILIC+**
L-Valine	**RP+ HILIC+ NMR**
Ergocalciferol	HILIC+
Xanthine	**RP+** HILIC-
